# Neural dynamics of mental state attribution to social robot faces

**DOI:** 10.1093/scan/nsaf027

**Published:** 2025-03-11

**Authors:** Martin Maier, Alexander Leonhardt, Florian Blume, Pia Bideau, Olaf Hellwich, Rasha Abdel Rahman

**Affiliations:** Department of Psychology, Humboldt-Universität zu Berlin, Berlin D-10099, Germany; Science of Intelligence, Research Cluster of Excellence, Technische Universität Berlin, Berlin D-10587, Germany; Department of Psychology, Humboldt-Universität zu Berlin, Berlin D-10099, Germany; Science of Intelligence, Research Cluster of Excellence, Technische Universität Berlin, Berlin D-10587, Germany; Computer Vision & Remote Sensing, Technische Universität Berlin, Berlin D-10587, Germany; Science of Intelligence, Research Cluster of Excellence, Technische Universität Berlin, Berlin D-10587, Germany; Inria, CNRS, Univ. Grenoble Alpes, Montbonnot-Saint-Martin 38330, France; Science of Intelligence, Research Cluster of Excellence, Technische Universität Berlin, Berlin D-10587, Germany; Computer Vision & Remote Sensing, Technische Universität Berlin, Berlin D-10587, Germany; Department of Psychology, Humboldt-Universität zu Berlin, Berlin D-10099, Germany; Science of Intelligence, Research Cluster of Excellence, Technische Universität Berlin, Berlin D-10587, Germany

**Keywords:** mind attribution, social robots, brain activity, social cognition, affective information

## Abstract

The interplay of mind attribution and emotional responses is considered crucial in shaping human trust and acceptance of social robots. Understanding this interplay can help us create the right conditions for successful human–robot social interaction in alignment with societal goals. Our study shows that affective information about robots describing positive, negative, or neutral behaviour leads participants (*N *= 90) to attribute mental states to robot faces, modulating impressions of trustworthiness, facial expression, and intentionality. Electroencephalography recordings from 30 participants revealed that affective information influenced specific processing stages in the brain associated with early face perception (N170 component) and more elaborate stimulus evaluation (late positive potential). However, a modulation of fast emotional brain responses, typically found for human faces (early posterior negativity), was not observed. These findings suggest that neural processing of robot faces alternates between being perceived as mindless machines and intentional agents: people rapidly attribute mental states during perception, literally seeing good or bad intentions in robot faces, but are emotionally less affected than when facing humans. These nuanced insights into the fundamental psychological and neural processes underlying mind attribution can enhance our understanding of human–robot social interactions and inform policies surrounding the moral responsibility of artificial agents.

## Introduction

The demand for social robots—embodied artificial systems designed for various uses such as care, retail, and entertainment—is expected to increase in the coming years, leading to more people engaging with them in both private and professional lives (Breazeal et al. 2016, Belpaeme et al. 2018, Paolillo et al. 2022). Yet, key psychological and neurocognitive aspects of interacting with social robots are still under investigation, including the extent to which humans—and their brains—process robots akin to intentional social agents with mental states (Marchesi et al. 2019, Henschel et al. 2020, Cross and Ramsey 2021). This question bears important implications for how we deal with artificial social agents as a society, including moral judgments of their responsibility for negative outcomes (Gray et al. 2012).

Previous theoretical work has proposed that two conflicting intuitions come into play, the ‘intentional’ stance and the ‘physical’ (or ‘design’) stance (Dennett 1989, Marchesi et al. 2019, Perez-Osorio and Wykowska 2020, Clark and Fischer 2023). Taking an intentional stance towards robots means to intuitively treat them as if they had a mind, attributing mental states like motivations, intentions, or emotions. This may enable people to engage processes that typically support social interaction with humans, such as theory of mind, as a basis for social perception, communication, and behavioural coordination (Dryer 1999, Epley et al. 2007, Gray and Wegner 2012, de Graaf 2016, Onnasch and Roesler 2019). On the other hand, people’s explicit opinions often reflect a physical stance towards robots, viewing them as machines designed or programmed to behave in specific ways (de Graaf et al. 2015, de Graaf 2016, Kim and Duhachek 2020, Clark and Fischer 2023, Jacobs et al. 2022).

How are these seemingly contradictory intuitions—robots as intentional beings or mindless machines—reflected in people’s perception of robots and the underlying neural processes? Most research has focused on how human–robot interaction and mind perception are influenced by robot design features (Duffy 2003, Waytz et al. 2010, Broadbent et al. 2013, Martini et al. 2016, Ceh and Vanman 2018, Hortensius et al. 2018, Onnasch and Roesler 2019) and perceiver traits, such as attitudes towards robots and artificial intelligence (AI) (Stafford et al. 2014, Spatola and Wudarczyk 2021, Jacobs et al. 2022, Thellman et al. 2022). However, crucial variables shaping social perception and interaction in real time have been largely overlooked in studies of mind attribution to robots: the human ability to perceive and evaluate others’ intentions is strongly influenced by context and prior knowledge, such as learned person-related information (Abdel Rahman 2011, Wieser et al. 2014, Otten et al. 2017, Baum et al. 2020, Maier et al. 2022). Our perception of others is not only shaped by what we can read from their faces, but also from what we read into them based on our expectations (Hassin and Trope 2000). For instance, social-affective information has been shown to influence brain signatures of early perceptual, reflexive emotional, as well as higher-level evaluative processing (Schacht and Sommer 2009, Abdel Rahman 2011, Wieser et al. 2014, Suess et al. 2015, Baum et al. 2020, Maier et al. 2022), as elaborated on below.

### The present study

**Figure 1.**
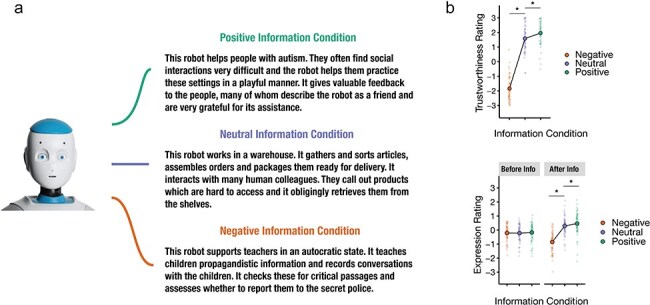
Information examples and rating results of Experiment 1. (a). Representative story examples for each affective information condition along with one example of a robot portrait. The pairings of robots and information type were counterbalanced across participants, ensuring each robot was paired equally as often with negative, neutral, and positive information. (b) Trustworthiness ratings after information acquisition and facial expression ratings before and after information acquisition, categorised by information condition. Large dots denote group means with corresponding 95% CIs, while small dots indicate individual participant means. Asterisks highlight statistically significant differences. Photo Romeo the Robot by Softbank Robotics Europe, 2016, Wikimedia Commons (https://commons.wikimedia.org/wiki/File:Romeo_the_robot_.jpg). CC BY 4.0. Cropped and rotated from original.

In this study, we investigate how brain dynamics reflect mental state attribution in the perception of social robots. Specifically, we test how prior information about robots’ behaviour influences neural correlates of perception, emotional responses, and evaluation (as illustrated in Fig. 1). In human social perception, prior knowledge or beliefs about others (e.g. ‘she bullied her work colleague’) can lead people to perceive emotional expressions in objectively neutral faces, revealing the attribution of mental states that are not necessarily present in the person (Abdel Rahman 2011, Suess et al. 2015, Maier et al. 2022). This can be attributed to the interplay between bottom-up and top-down processing, where perceptions are constructed based on combinations of sensory input from the environment and predictions generated from prior knowledge and expectations (Lee and Mumford 2003, Friston 2009, Clark 2013, Press and Yon 2019, Lupyan et al. 2020, Maier et al. 2022). Here we investigate this mechanism for the first time with robot faces: does information about a robot’s behaviour literally make people see good or bad intentions in its face, indicating mind attribution at an early perceptual stage? And do people react emotionally to neutral robot faces based on whether they are associated with morally good or bad behaviour, as they would with other humans (Abdel Rahman 2011, Suess et al. 2015, Baum et al. 2020, Baum and Abdel Rahman 2021)?

In two preregistered experiments, participants were presented with positive, neutral, and negative information about real human-like robots (e.g. ‘teaches social skills to people with autism’, ‘assembles orders at a warehouse’, ‘reports children to the secret police’; see Fig. 1). Subsequently, they rated the valence of the robots’ facial expressions and their trustworthiness. We hypothesised that, similar to human faces, participants would attribute emotional expressions to the robots’ faces and assess their trustworthiness influenced by the information they had acquired. Experiment 1 investigated the effect of affective information on ratings of facial expression and trustworthiness, seeking initial evidence that people indeed read good or bad intentions into robot faces. To explore the neurocognitive dynamics associated with the attribution of mental states, Experiment 2 measured evoked brain responses using electroencephalography (EEG) as participants evaluated robots’ facial expressions.

## Materials and methods

### Experiment 1

The preregistration for Experiment 1 can be accessed at https://osf.io/qytra.

#### Participants

Sixty participants (22 cisgender women, 38 cisgender men; mean age 28 years, range 18–39) were recruited from Prolific (prolific.com) and received monetary compensation. Thirty participants were German speakers (6 cisgender women, 24 cisgender men; mean age 28 years, range 19–39), and 30 were English speakers (16 cisgender women, 14 cisgender men; mean age 27 years, range 18–37). The sample size, a multiple of six ensured counterbalancing across three information conditions and two orders of response button assignments (see Supplementary Material for exclusion criteria). The study adhered to the principles of the Declaration of Helsinki and received approval from the Ethics Committee of the Department of Psychology at Humboldt-Universität zu Berlin. Participants provided informed written consent before participation.

#### Materials

The picture stimuli comprised 36 full-colour frontal portrait photographs featuring existing humanoid robots, each displaying approximately neutral facial expressions (refer to Fig. 1 for an example; further details including names and sources of the robot images are listed in the Supplementary Material). Robot heads were cropped and placed on a grey background and matched in size and eye placement across all images. All robot images had frontal gaze.

We recorded 36 spoken stories in both English (mean duration: 18.1 s) and German (mean duration: 17.9 s) with affectively positive, neutral, or negative information about the robots (see Fig. 1 for examples and Supplementary Material for all stories). To ensure that the stories would be perceived as positive, neutral, or negative according to the respective condition, we pretested them with a separate sample of participants (*N* = 15). The valence and arousal ratings of the stories were as expected. Additionally, positive and negative stories were rated as equally realistic, while neutral stories were rated as more realistic compared to both positive and negative stories. Detailed prerating results are provided in the Supplementary Material.

#### Procedure

Participants initially rated the facial expressions of 36 robots on a 7-point Likert scale ranging from very negative to very positive. The scale’s midpoint was neutral, and the order of anchors counterbalanced across participants. In the main section, participants were presented with six robots per block, each paired with a different story (two of each information condition). They rated each robot’s trustworthiness on a 7-point Likert scale immediately after hearing the story and subsequently rated the facial expressions of all robots in the block. Attention was monitored with multiple-choice questions between blocks. After the main experiment, participants completed the Attitude towards Artificial Intelligence Scale (Sindermann et al. 2021) and answered questions about the experiment. They reported whether they had researched information about the robots, were distracted, or distrusted any of the presented information, and rated the robots’ perceived intentionality and deliberateness. Those who distrusted the information estimated the percentage of stories affected. Participants also provided feedback and were debriefed that none of the information was related to the featured robots.

### Experiment 2

Experiment 2 was preregistered under https://osf.io/c8va7. The procedure and materials were similar to Experiment 1, with the following differences due to the EEG setting: (i) Experiment 2 was divided into two main parts: a learning phase (without EEG) in which participants acquired and rehearsed information about all robots, and a subsequent EEG part in which participants performed rating tasks on the robot pictures; (ii) only a subset of 18 robot stimuli was used to reduce the length of the experiment and information to memorise; (iii) rating scales were 5-point instead of 7-point-Likert scales due to the experimental setup in the EEG laboratory; (iv) trustworthiness ratings were collected both before and after learning, whereas facial expression ratings were only collected after learning; and (v) a new task was added at the end to collect perceived intentionality ratings for each robot.

#### Participants

Thirty-five participants, all German speakers, were tested to achieve the preregistered final sample of 30 participants (24 cisgender women, 6 cisgender men; mean age 25.4 years, range 18–36). During debriefing, the five excluded participants had expressed strong doubts about the veracity of the information provided about the robots (see Supplementary Material for power analysis and exclusion criteria). All participants received monetary compensation or course credit. The study adhered to the principles of the Declaration of Helsinki and received approval from the Ethics Committee of the Department of Psychology at Humboldt-Universität zu Berlin. Participants provided written consent before participating.

#### Materials

A subset of 18 robot pictures with the corresponding stories was selected from the stimulus set of Experiment 1 (see Table S2 in the Supplementary Material). Stimuli were presented on a grey background on a 19-inch LCD monitor with a resolution of 1280 × 1024 pixels and a 75 Hz refresh rate. During the rating tasks, robot faces were displayed with a size subtending 6.03° vertical and 6.02° horizontal visual angles (viewing distance: 70 cm). We recorded additional short versions of each robot story, focusing on a central part of the robots’ behaviour. After one presentation of each story’s long version, further repetitions used the short versions.

The added perceived intentionality rating was inspired by the InStance questionnaire (Marchesi et al. 2019). Participants rated the robots’ behaviour on a scale from −50 (mechanistic) to 50 (intentional), indicating the extent to which they agreed with mechanistic versus intentional descriptions of the robot’s behaviour. Details on the intentionality questionnaire are provided in the Supplementary Material.

#### Procedure

**Figure 2.**
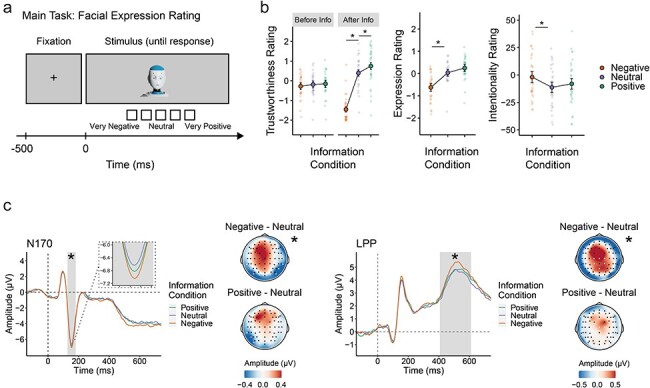
EEG study results. (a). Trial sequence of the facial expression rating task. (b) Trustworthiness ratings before and after information acquisition, facial expression and intentionality ratings after information acquisition, categorised by information condition. Large dots denote group means with corresponding 95% CIs, while small dots indicate individual participant means. (c) Grand average ERPs for the N170 and LPP components collected during the facial expression rating task. Grey shading highlights time windows for the N170 and LPP. Scalp topographies illustrate differences between the information conditions, with channels included in the N170 and LPP regions of interest highlighted in white. Asterisks highlight statistically significant differences. Photo Romeo the Robot by Softbank Robotics Europe, 2016, Wikimedia Commons (https://commons.wikimedia.org/wiki/File:Romeo_the_robot_.jpg). CC BY 4.0. Cropped and rotated from original.

The experiment began with participants rating each robot’s trustworthiness. Following this, participants entered a 30-min learning phase, during which they acquired and rehearsed information about 18 robots (details provided in the Supplementary Material). EEG electrodes were placed after the learning phase. Participants then rated the trustworthiness of the robots again, followed by facial expression and intentionality ratings. The facial expression task was repeated 12 times for each robot in random order. This task formed the basis for the analysis of event-related potentials (ERPs). As shown in Fig. 2, each trial began with a fixation cross displayed for 500 ms, followed by the robot face stimulus, which remained visible until participants responded. To ensure accurate use of the five response buttons corresponding to the rating scale, participants were reminded of the scale at regular intervals during breaks and had a printed reference sheet on the table. The placement of scale anchors (e.g. very positive, very negative) was counterbalanced across participants.

#### EEG recording and analysis

The EEG data were acquired using Ag/AgCl electrodes placed at 64 scalp sites according to the extended 10–20 system, with a sampling rate of 500 Hz and all electrodes referenced to the left mastoid. The electrooculogram (EOG) was recorded using a bipolar vertical EOG channel consisting of electrodes Fp1—IO1 and a bipolar horizontal EOG channel consisting of electrodes F9—F10. During recording, a low-cut-off filter (0.032 Hz) was applied, and electrode impedances were maintained below 10 kΩ. Post-experiment, a calibration procedure was conducted to capture prototypical eye movements for subsequent artefact correction.

Offline processing for single-trial ERP analysis followed a pipeline detailed in Frömer et al. (2018), reimplemented using functions of MNE Python (Gramfort), available at https://github.com/alexenge/hu-neuro-pipeline. Continuous EEG data were rereferenced to a common average reference, and eye movement artefacts were removed using a spatio-temporal dipole modelling procedure with the BESA software (Ille et al. 2002). The corrected data were low-pass filtered at 40 Hz, segmented into epochs of −500 to 1500 ms relative to face stimulus onset, and baseline-corrected using the 200 ms prestimulus interval. For each participant, 72 segments were created in each information condition (six robots per information condition ✕ 12 repetitions), excluding segments containing artefacts (amplitudes over ±150 µV, or changing by more than 50 µV between samples). After artefact rejection, over 99% of segments remained per condition, leaving *M* = 71.37 segments per subject in the negative condition (*SD* = 1.25; range = 67–72), *M* = 71.33 segments in the neutral condition (*SD* = 1.06; range = 68–72), and M = 71.37 segments in the positive condition (*SD* = 1.16; range = 68–72). These represent the trials that were analysed and are reported in the EEG results.

Single-trial mean amplitudes were obtained for the P1, N170, early posterior negativity (EPN), and late positive potential (LPP) components by averaging across preregistered time windows and electrode sites typical for each component (see Fig. 2) and analysed using linear mixed effects models (LMMs). The P1 was averaged at parieto-occipital electrode sites (O1, O2, Oz, PO7, PO8) in the time window 76–116 ms centered around the average P1-peak of the ERP collapsed across all conditions. The N170 was averaged at parieto-occipital electrode sites (TP9, TP10, P7, P8, PO9, PO10, O1, O2) centered around its average peak, between 129 and 179 ms. The EPN was averaged at posterior electrodes (PO7, PO8, PO9, PO10, TP9, TP10) between 220 and 350 ms. The LPP was averaged at centro-parietal sites (Pz, Cz, C1, C2, CP1, CP2) between 408 and 608 ms.

## RESULTS

### Experiment 1

In Experiment 1, an online study investigated the influence of robot-related information on trustworthiness and facial expression ratings in 60 participants, evenly split between English and German native speakers. After the information manipulation, we anticipated both trustworthiness and facial expression ratings to align with the valence of the learned information. Rating data were analysed using LMMs with fixed effects coded as sliding difference contrasts (see Supplementary Material for details).

#### Facial expression ratings

As expected, before participants had learned information about the robots, facial expression ratings were neutral overall and showed no significant differences between conditions (see Table 1, Fig. 1). However, after hearing the stories, participants rated the same facial expressions differently depending on the valence of the associated information. Facial expressions in the negative condition were rated as more negative than in the neutral condition and facial expressions in the positive condition were rated as more positive than in the neutral condition. An additional analysis of variance confirmed the significance of the interaction between phase and information, *F*(4, 59.53) = 24.16; *P* < .001, showing that the overall differences between the information conditions increased significantly between phases. An analysis with phase nested within information showed that shifts in expression ratings from pre- to post-learning were nearly identical in the negative (∆ = −0.64) and positive (∆ = 0.63) conditions (cf. Fig. 2B). The neutral condition showed a significant positive shift (∆ = 0.51). Detailed results are provided in the Supplementary Material. An additional LMM analysis including the variable language indicated no differences in the facial expression ratings provided by English and German speakers (details in Supplementary Material).

**Table 1.** Facial expression rating results

Predictors	*b*	95% CI	*P*-value
Intercept	−0.12	[−0.35, 0.12]	.326
Phase(Post-Pre)	0.17	[0.10, 0.24]	**<.001**
Phase(Pre):Information(Neu-Neg)	−0.01	[−0.13, 0.11]	.873
Phase(Post):Information(Neu-Neg)	1.14	[1.02, 1.26]	**<.001**
Phase(Pre):Information(Pos-Neu)	0.05	[−0.07, 0.17]	.437
Phase(Post):Information(Pos-Neu)	0.17	[0.05, 0.29]	.**005**
Random Effects			SD
Participants			0.19
Stimuli			0.37
Residual			1.21
Deviance	13 764.60		
Log-Likelihood	−6882.30		

Results of linear mixed model analyses of Facial Expression Ratings in Experiment 1. *Note*. Information Conditions: Neg = Negative, Neu = Neutral, Pos = Positive; Phase Conditions: Pre = Pre-learning, i.e. before information acquisition, Post = Post-learning, i.e. after information acquisition; colons indicate nesting of fixed variables; boldface indicates statistical significance at α = 0.05.

#### Trustworthiness ratings

Analysis of postlearning trustworthiness ratings yielded a main effect of information. As expected, participants rated robots as significantly more trustworthy when matched with positive information compared to neutral information. Robots matched with negative information were rated as less trustworthy in comparison to robots matched with neutral information (see Table 2, Fig. 1). An additional analysis including the independent variable group (English vs. German speakers) revealed a main effect of group on trustworthiness ratings (b = 0.30, 95% CI = [0.01, 0.59], *P* = .041). Overall, English-speaking participants rated the robots’ trustworthiness slightly higher than German-speaking participants (details in Supplementary Material).

**Table 2.** Trustworthiness rating results

Predictors	*b*	95% CI	*P*-value
Intercept	0.57	[0.40, 0.74]	**<.001**
Information(Neu-Neg)	3.42	[3.15, 3.70]	**<.001**
Information(Pos-Neu)	0.37	[0.22, 0.53]	**<.001**
Random Effects			SD
Participants			0.55
Information(Neu-Neg)			0.97
Information(Pos-Neu)			0.43
Stimuli			0.25
Residual			1.03
Deviance	6606.81		
Log-Likelihood	−3303.41		

Results of linear mixed model analyses of Trustworthiness Ratings in Experiment 1. *Note*. Information Conditions: Neg = Negative, Neu = Neutral, Pos = Positive. Boldface indicates statistical significance at α = 0.05.

#### Discussion of Experiment 1

In Experiment 1, we observed differential effects of affective information on facial expression ratings, providing initial evidence that learned information may lead humans to perceive emotional facial expressions in robot faces. Notably, in the absence of prior information, these faces were initially rated as neutral. These findings imply that participants attributed mental states during robot face perception. Additionally, the affective information manipulation influenced participants’ trustworthiness ratings, demonstrating that people clearly distinguish between the trustworthiness of robots described as fulfilling negative, neutral, and positive tasks. The additional main effect of participant group on trustworthiness ratings may reflect genuine differences in trust evaluation among English and German speakers. Alternatively, despite our efforts to maintain consistency in meaning across languages, it could stem from nuances in the robots’ backstories conveyed differently in each language.

To account for potential task demand effects in facial expression ratings, Experiment 2 used EEG to explore the underlying neurocognitive mechanisms. ERPs were employed to directly assess the impact of information on perception, with early ERP components being less influenced by task demands.

### Experiment 2

In Experiment 2, we used EEG to investigate the neural mechanisms and temporal dynamics underlying the attribution of mental states to robot faces. We tested a new sample of 30 native German speakers, using the same information manipulation for a subset of 18 out of the 36 robots from Experiment 1.

Using ERPs, we investigated distinct stages of processing in the brain with high temporal precision, aiming to determine which stages support mental state attribution to robots. Our analysis focused on early visual perception (P1 component; Di Russo et al. 2002, Haynes et al. 2003), visual processing of faces and facial expressions (N170 component; Bentin et al. 1996, Eimer et al. 2010), fast reflexive emotional responses (EPN; Schupp et al. 2006, Schacht and Sommer 2009), and more elaborate stimulus evaluation (LPP; Schupp et al. 2006, Baum et al. 2020).

To better understand the relationship between affective information, ERP components, and intentionality, we added a rating task to assess the intentionality attributed to each robot’s behaviour and examine whether affective information enhances perceived intentionality. We also explored the link between specific ERP components and the likelihood of attributing intentionality to robots, considering the intentionality rating as a covariate.

#### Rating results

Facial expression ratings were collected after the learning part. Each robot’s facial expression was rated 12 times. We report results based on the first rating of each stimulus. Facial expression ratings were again influenced by affective information: expressions of robots associated with negative information were rated as more negative than those associated with neutral information (*b* = 0.69, 95% CI = [0.40, 0.97], *P* < .001). Expressions of robots in the positive information condition were rated as slightly more positive compared to the neutral information condition, but this comparison only yielded a statistical trend (*b* = 0.18, 95% CI = [−0.01, 0.37], *P* = .069).

Trustworthiness ratings were collected before and after learning. In the baseline ratings before learning, no significant differences were found between the robots assigned to the three information conditions (neutral—negative: *b* = 0.09, 95% CI = [−0.18, 0.35], *P* = .506; positive—neutral: *b* = 0.03, 95% CI = [−0.19, 0.25], *P* = .802). However, after learning, significant differences emerged (neutral—negative: *b* = 1.83, 95% CI = [1.57, 2.10], *P* < .001; positive—neutral: *b* = 0.33, 95% CI = [0.11, 0.55], *P* = .003). An additional analysis of variance confirmed the significance of the interaction between phase and information, *F*(2, 973.78) = 114.95; *P* < .001, showing that the overall differences between trustworthiness ratings in the different information conditions increased significantly between phases. Taken together, the results of Experiment 2 align well with those obtained in Experiment 1.

Intentionality ratings, assessed on a scale from −50 (non-intentional) to 50 (intentional), yielded a mean of −7.05 (95% CI = [−13.34, −0.69]), indicating a tendency towards nonintentional explanations, though not entirely so. Analysis showed that robots associated with negative information were rated as more intentional than those with neutral information (*b* = −9.67, 95% CI = [−15.62, −3.73], *P* < .001). No significant difference was found between neutral and positive information (*b* = 3.19, 95% CI = [−2.76, 9.14], *P* = .293). This suggests that framing robots with negative behaviour increases perceived intentionality. Overall, affective information influenced ratings of facial expression, trustworthiness, and perceived intentionality.

#### EEG results

We tested the effects of negative, neutral, and positive information on the processing of robot faces presented during the facial expression rating task. Specifically, we analysed ERP components associated with early perceptual processing (P1 and N170), reflexive emotional responses to visual input (EPN), and higher-level evaluation (LPP).

We observed significant influences of affective information on the N170 and LPP components, but not the P1 and EPN components (see Tables 3 and 4 and Fig. 2). Both N170 and LPP amplitudes were significantly increased in the negative information condition compared to the neutral information condition. There were no significant differences between the neutral and the positive information conditions.

**Table 3.** Perception-related EEG results

	P1 component			N170 component		
Predictors	*b*	95% CI	*P*-value	*b*	95% CI	*P*-value
Intercept	3.11	[1.79, 4.43]	**<.001**	−5.22	[−6.53, −3.92]	**<.001**
Information(Neu-Pos)	−0.12	[−0.43, 0.19]	.455	0.21	[−0.06, 0.47]	.127
Information(Neg-Neu)	0.10	[−0.21, 0.42]	.524	−0.34	[−0.60, −0.08]	.**012**
Random Effects			SD			SD
Participants			3.45			3.35
Stimuli			0.59			0.82
Residual			5.24			4.42
Deviance	39 670.08			37 501.78		
log-Likelihood	−19 835.04			−18 750.89		

Results of linear mixed model analyses of the P1 and N170 components. *Note*. Information Conditions: Neg = Negative, Neu = Neutral, Pos = Positive. Boldface indicates statistical significance at α = 0.05.

**Table 4.** Emotion-related EEG results

	EPN			LPP		
Predictors	*b*	95% CI	*P*-value	*b*	95% CI	*P*-value
Intercept	−1.02	[−2.38, 0.33]	.135	4.35	[3.60, 5.10]	**<.001**
Information(Neu-Pos)	−0.06	[−0.34, 0.21]	.657	−0.16	[−0.48, 0.16]	.322
Information(Neg-Neu)	−0.19	[−0.46, 0.09]	.181	0.48	[0.18, 0.78]	.**003**
Random Effects			SD			SD
Participants			3.35			1.94
Information(Neu-Neg)						0.447
Information(Pos-Neu)						0.36
Stimuli			1.14			0.35
Residual			4.60			4.45
Deviance	38 020.26			37 543.59		
log-Likelihood	−19 010.13			−18 771.79		

Results of linear mixed model analyses of the EPN and LPP components. *Note*. Information Conditions: Neg = Negative, Neu = Neutral, Pos = Positive. Boldface indicates statistical significance at α = 0.05.

We examined whether perceived intentionality was linked to the impact of information on the N170 and LPP components by calculating an additional LMM for each, using centered intentionality scores as a covariate. We found an interaction between intentionality and information (negative—neutral) in the N170 component (*b* = −0.29, 95% CI = [−0.57, −0.01], *P* = .045), with higher intentionality scores associated with a greater effect of negative information on N170 amplitudes. In the LPP, higher intentionality scores were linked to lower LPP amplitudes (*b* = −0.14, 95% CI = [−0.28, −0.01], *P* = .033).

We conducted two additional control analyses, detailed in the Supplementary Material. To rule out task demand effects, we included participants’ scores on the Perceived Awareness of the Research Hypothesis Scale (Rubin 2016) as a covariate in the analysis of expression ratings from Experiment 2. This analysis confirmed that the main effect of negative knowledge remained robust. A trend was observed for an interaction between hypothesis awareness and the negative knowledge effect, whereby participants with the least awareness of the hypothesis tended to exhibit the strongest knowledge effects. These findings suggest that social desirability or explicit demand characteristics did not significantly influence the results. If anything, participants aware of the hypothesis might have actively avoided giving biased responses, countering concerns about social desirability. Additionally, we tested whether differences in story realism ratings (neutral stories were rated as more realistic than positive and negative ones during prerating) influenced EEG or expression rating results by including each story’s realism rating as a covariate. This analysis revealed no main effects of realism or interactions with knowledge effects on ERP components (N170, EPN, LPP) or facial expression ratings (see Supplementary Material for details).

#### Discussion of Experiment 2

In line with the findings of Experiment 1, the affective information manipulation significantly influenced trustworthiness and facial expression ratings. We also observed an impact of affective information on perceived intentionality, indicating that robots were judged as more intentional when they were linked to negative backstories. This corresponds with the asymmetry often noted in judgments of human behaviour, where people tend to attribute greater intentionality for actions with negative outcomes compared to positive ones (Knobe 2003, Feltz 2007).

In ERPs, we observed significant information effects on two distinct processing stages: the N170 component, associated with visual perception, and the later LPP component, reflecting more elaborate stimulus evaluation. The N170 is most commonly associated with structural visual encoding of faces, and it has been shown to be sensitive to manipulations of facial expression, as well as the realism of face images (Bentin et al. 1996, Schindler et al. 2017, Schindler and Bublatzky 2020). Thus, robot faces associated with negative information may be perceived as displaying an emotional facial expression. Alternatively, since robot faces represent intermediate stimuli between faces and objects (Geiger and Balas 2021), affective knowledge could shift their perception towards more ‘face-like’ processing. Both interpretations support the idea that affective information facilitates attribution of mental states to robot faces at an early, potentially automatic perceptual stage. The differentiation of the N170 for identical robot faces based solely on associated affective information represents, to our knowledge, a novel finding. This highlights how top-down influences, such as semantic or affective knowledge, can modulate early visual components (P1 and N1/N170). Such effects have been previously documented for other stimulus categories, including human faces (Righart and de Gelder 2006, Luo et al. 2016), objects (Maier et al. 2014, Maier and Abdel Rahman 2019, 2024, Enge et al. 2023), and colours (Thierry et al. 2009, Maier and Abdel Rahman 2018).

The information effect on the LPP indicates that negatively framed robots are evaluated as more emotionally relevant compared to neutrally framed robots (Abubshait and Wiese, Schupp et al. 2006). Affective information did not influence the P1 component, indicating that low-level visual processing remained unaffected. Since previous studies have demonstrated knowledge effects in the P1 for objects (Abdel Rahman and Sommer 2008, Maier et al. 2014), this suggests that the visual processing of robot faces leaned more towards face perception rather than object perception.

Notably, contrary to our prediction, affective information did not influence the EPN component. This suggests that the modulation of early emotional responses typically induced by affective information for human faces (e.g. Abdel Rahman 2011, Suess et al. 2015, Luo et al. 2016, Baum and Abdel Rahman 2021, Ziereis and Schacht 2023) is absent or attenuated for robot faces. We propose this stems from meaningful differences in social perception between human and robot faces, suggesting that processing of robots tends to be more visually driven and less emotionally resonant. This point is elaborated in the general discussion. Still, other factors might explain why N170 but not EPN effects were observed. For example, the N170 is known to be particularly sensitive to the eyes (Itier et al. 2007), whereas the EPN appears to be sensitive to the mouth region (Langeslag et al. 2018). Given that many robot stimuli in our study featured large, prominent eyes but small or absent mouths, the design characteristics of the robots may have contributed to this dissociation. Future studies should investigate how specific facial features of robots interact with affective context information. Additionally, the highly stylised nature of the robot faces in our study may have played a role. Schindler et al. (2017) reported differences in N170 localisation between cartoon and realistic faces, suggesting that cartoon faces rely more on structural analysis associated with the occipital face area, while realistic faces engage holistic processing in the fusiform face area. This may explain why we observed a modulation of the N170, as the processing of stylised faces seems to rely heavily on lower-level visual processes. However, stylisation alone cannot fully account for our findings. Schindler *et al*. also showed that the effect of emotional expressions on the EPN does not differ between stylised and realistic human faces, indicating that stylisation alone cannot explain the absence of an EPN effect in our study.

The analysis including perceived intentionality as a covariate allowed us to explore the relationship between affective information, intentionality, and the neurocognitive processes reflected in the N170 and LPP components. The interaction between affective information and perceived intentionality on the N170 further links the perceptual bias caused by negative information to the attribution of intentionality. Two interpretations are plausible: visual face processing enhanced by negative information may increase mental state attribution, or robot faces inherently more predisposed to attribution of intentionality may be more likely to have emotional expressions inferred onto them. This open question warrants further exploration in future research. Moreover, higher perceived intentionality scores were associated with a reduction in LPP amplitudes, independent of affective information. This may indicate that the cognitive effort required for emotional evaluation decreases for robot faces perceived as more intentional (Matsuda and Nittono 2015, Eiserbeck et al. 2023). Robot faces more easily attributed with intentionality might require less deliberation during social-emotional processing, suggesting a more efficient use of theory of mind.

## General discussion

Humanoid social robots present an intriguing puzzle: people often intuitively interact with robots as they would with another human, even though they may be explicitly aware that robots are mechanical artefacts that do not share the same cognitive abilities as humans (de Graaf 2016, Clark and Fischer 2022). Thus, people may perceive robots through a ‘physical’ or ‘intentional’ lens, sometimes attributing mechanical causation to their behaviour, and at other times, mental causation. In this study, we explored how these shifting perspectives manifest during the perception of robot faces, focusing on the extent to which brain processing reveals the attribution of mental states.

Experiment 1 established that the valence of information about robots’ behaviour influenced people’s ratings of robots’ trustworthiness and facial expressions. The same robots were distrusted when associated with negative information (e.g. the robot reports children to the secret police), but were trusted when associated with positive information (e.g. the robot teaches social skills to people with autism). Crucially, participants also rated the objectively neutral facial expressions of robots as more negative when paired with negative information and as more positive when paired with positive information, compared to neutral information. These results provided initial evidence that people in fact read good or bad intentions into robot faces—an effect previously observed only during the perception of human faces (Abdel Rahman 2011, Suess et al. 2015).

Experiment 2 showed that affective information influenced brain dynamics underlying the attribution of mental states to robot faces, affecting both perceptual encoding (N170 ERP component) and more elaborate stimulus evaluation (LPP component). The modulation of the N170 highlights the speed of mental state attribution: changes to perceptual processing occur within 130 to 180 ms, at a stage that typically precedes conscious access (Förster et al. 2020). This effect suggests that people not only evaluate robot faces differently, but literally see bad intentions in a robot face associated with negative behaviour. The later effect of information on the LPP indicates that negatively framed robots are also evaluated as more emotionally relevant. Interestingly, contrary to our prediction, affective information did not influence the EPN during robot face processing, indicating the absence of an early, reflexive emotional response typically elicited by similar affective information in the processing of human faces (Abdel Rahman 2011, Suess et al. 2015). The notion that affective responses are less malleable in response to robots compared to humans is supported by fMRI-evidence: Chaminade et al. (2010) found that the perception of emotions expressed by a humanoid robot engaged brain regions associated with visual processing (occipital and posterior temporal cortices) more strongly than those associated with emotional resonance, such as the anterior insula and orbitofrontal cortex. Similarly, brain activity linked to social bonding does not increase over prolonged interaction with robots, as it does in human–human interaction (Spatola and Chaminade 2022). Together, these findings suggest partly distinct mechanisms underlying social perception of robots, emphasising visual processing rather than reflexive emotional arousal.

Our exploratory analyses uncovered further connections between perceived intentionality, affective information, and brain responses. Firstly, robots were perceived as more intentional when paired with negative rather than neutral information, suggesting that framing robots with emotional background information enhances the attribution of mental causes for their actions. Secondly, the perceived intentionality of individual robots was associated with the impact of negative information on the N170 component. This correlation further underscores the idea that the intentionality we attribute to a robot face based on affective information is supported by visual processes, as something we automatically perceive in its facial expression.

Taken together, it appears that both the intentional and physical stance are reflected in brain dynamics, but at different processing stages. In line with the intentional stance, we rapidly and automatically read mental states into robot faces during visual perception (N170). We also explicitly evaluate robots in the light of acquired information, as shown in ratings and the LPP. However, in our fast emotional reaction (EPN), we are not as affected as we would be by comparable negative information about other humans. This suggests that the brain’s emotional response to humanoid robots is influenced more by the physical stance than by social or intentional aspects. In conclusion, while we do engage perceptual and cognitive aspects of social cognition to process social robots, our findings on emotional processing suggest that we do not experience them as fully fledged intentional and social agents.

### Implications for social robotics and policy

Our results emphasise that acceptance and moral judgments of social robots hinge on the interplay of intentionality and emotional valence. Emotional information can increase perceived intentionality, both on the level of explicit ratings and automatic perceptual processing in the brain. Intentionality plays a key role in moral judgment, both in that mindedness may be a prerequisite for moral responsibility and that moral transgressions may necessitate intentional agency (Killen et al. 2011, Decety and Cacioppo 2012, Gray et al. 2012, Abubshaitand Wiese ; Wiese et al. 2017).

Although humanoid robots lack actual intentionality, our results show that contextual semantic and emotional cues can induce perceptions of intentionality, potentially leading to moral judgments based on these attributions. If robots are perceived as more intentional when associated with negative actions, they may be inappropriately judged as morally responsible. This raises concerns about ‘responsibility gaps,’ where no party is properly held accountable for harmful outcomes caused by artificial agents (Matthias 2004, Sparrow 2007, Bryson 2018, Bigman et al. 2019). Thus, the effects of psychological variables like users’ beliefs and contextual affective information should be considered in policies legislating moral responsibility when deploying artificial social agents.

## Conclusion

We discovered that humans rapidly attribute mental states to humanoid robots following exposure to affective information about the robots’ behaviour. This was observed in brain dynamics reflecting both perceptual processing and more deliberate evaluation of robot faces, but notably absent during fast emotional processing, which lacked a component seen in the social perception of humans. These findings imply that the processing of social robots oscillates between being perceived as mindless machines and intentional agents, contingent upon the stage of perceptual and emotional processing in the brain. Such nuanced insights into the neural, cognitive, and affective mechanisms underlying the perception of robots have significant implications for social robotics, including policies regarding the moral responsibility of artificial agents. These considerations are especially pertinent given the projected proliferation of such agents in our societies.

## Supplementary Material

nsaf027_Supp

## Data Availability

The data and analysis code that support the findings of this study are available upon publication at https://osf.io/5bj7x.
