# Supplementary material for: Neural dynamics of mental state attribution to social robot faces
Source: Soc Cogn Affect Neurosci. 2025 Mar 11;20(1):nsaf027. doi: 10.1093/scan/nsaf027 (PMC11969468; doi:10.1093/scan/nsaf027)
Supplement: nsaf027_Supp [file nsaf027_supp.zip › scan-24-286-File020.docx]

**Table S10. Shifts in Facial Expression Rating Results Before vs. After Knowledge Acquisition.** Linear mixed model analysis of facial expression ratings in Experiment 1, with the independent variable Phase (pre- vs. post-knowledge acquisition) nested within the independent variable Information (negative, neutral, positive).

| Predictors | *b* | 95% CI | *p*-value |
| --- | --- | --- | --- |
| Intercept | -0.12 | [-0.35, 0.12] | 0.324 |
| Information(Neu-Neg) | 0.57 | [0.43, 0.70] | **<0.001** |
| Information(Pos-Neu) | 0.11 | [0.01, 0.20] | **0.026** |
| Information (Neg):Phase(Post-Pre) | -0.64 | [-0.76, -0.52] | **<0.001** |
| Information (Neu):Phase(Post-Pre) | 0.51 | [0.39, 0.63] | **<0.001** |
| Information (Pos):Phase(Post-Pre) | 0.63 | [0.52, 0.75] | **<0.001** |
| Random Effects |  |  | *SD* |
| Participants |  |  | 0.44 |
| Information(Neu-Neg) |  |  | 0.31 |
| Information(Pos-Neu) |  |  | 0.05 |
| Stimuli |  |  | 0.61 |
| Information(Neu-Neg) |  |  | 0.19 |
| Information(Pos-Neu) |  |  | 0.11 |
| Residual |  |  | 1.14 |
| Deviance | 13728.06 |  |  |
| log-Likelihood | -6864.03 |  |  |

Note. Information Conditions: Neg = Negative, Neu = Neutral, Pos = Positive; Phase Conditions: Pre = pre-learning, i.e. before information acquisition, Post = post-learning, i.e. after information acquisition; Colons indicate nesting of fixed variables; Boldface indicates statistical significance at α = .05.
