# Supplementary material for: Neural dynamics of mental state attribution to social robot faces
Source: Soc Cogn Affect Neurosci. 2025 Mar 11;20(1):nsaf027. doi: 10.1093/scan/nsaf027 (PMC11969468; doi:10.1093/scan/nsaf027)
Supplement: nsaf027_Supp [file nsaf027_supp.zip › scan-24-286-File021.docx]

**Table S11. Covariate Analyses including Story Realism.** Results of analyses for facial expression ratings and ERP components (N170, EPN, LPP) with independently rated realism scores for each story included as a covariate.

|  | **Facial Expression Ratings** | | | |  | | **N170** | | | |  | | **EPN** | |  |  | | **LPP** | | |  |
| --- | --- | --- | --- | --- | --- | --- | --- | --- | --- | --- | --- | --- | --- | --- | --- | --- | --- | --- | --- | --- | --- |
| Predictors | *b* | 95% CI | *p*-value |  | | *b* | | 95% CI | *p*-value |  | | *b* | | 95% CI | *p*-value |  | *b* | | 95% CI | *p*-value | |
| Intercept | -0.15 | [-0.43, 0.13] | 0.286 |  | | -5.23 | | [-6.54, -3.92] | **<0.001** |  | | -1.12 | | [-2.48, 0.24] | 0.104 |  | 4.40 | | [3.64, 5.16] | **<0.001** | |
| Information(Neu-Neg) | 0.77 | [0.39, 1.14] | **<0.001** |  | | 0.22 | | [-0.22, 0.66] | 0.337 |  | | 0.13 | | [-0.33, 0.59] | 0.568 |  | -0.50 | | [-0.93, -0.07] | **0.024** | |
| Information(Pos-Neu) | 0.20 | [-0.01, 0.42] | 0.067 |  | | -0.13 | | [-0.44, 0.17] | 0.388 |  | | 0.23 | | [-0.09, 0.55] | 0.153 |  | 0.09 | | [-0.21, 0.39] | 0.559 | |
| Real | 0.02 | [-0.13, 0.17] | 0.772 |  | | 0.03 | | [-0.18, 0.23] | 0.803 |  | | 0.13 | | [-0.09, 0.34] | 0.250 |  | -0.12 | | [-0.31, 0.08] | 0.236 | |
| Information(Neu-Neg) × Real | 0.18 | [-0.17, 0.53] | 0.311 |  | | 0.03 | | [-0.46, 0.52] | 0.910 |  | | 0.39 | | [-0.12, 0.91] | 0.134 |  | -0.22 | | [-0.69, 0.25] | 0.351 | |
| Information(Pos-Neu) × Real | 0.13 | [-0.14, 0.40] | 0.335 |  | | -0.19 | | [-0.57, 0.18] | 0.318 |  | | 0.07 | | [-0.32, 0.46] | 0.724 |  | -0.28 | | [-0.64, 0.09] | 0.139 | |
| Random Effects |  |  | *SD* |  | |  | |  | *SD* |  | |  | |  | *SD* |  |  | |  | *SD* | |
| Participants |  |  | 0.26 |  | |  | |  | 3.35 |  | |  | |  | 3.35 |  |  | |  | 1.94 | |
| Information(Neu-Neg) |  |  | 0.55 |  | |  | |  |  |  | |  | |  |  |  |  | |  |  | |
| Information(Pos-Neu) |  |  | 0.04 |  | |  | |  |  |  | |  | |  |  |  |  | |  |  | |
| Stimuli |  |  | 0.38 |  | |  | |  | 0.83 |  | |  | |  | 1.14 |  |  | |  | 0.35 | |
| Residual |  |  | 0.91 |  | |  | |  | 4.42 |  | |  | |  | 4.60 |  |  | |  | 4.45 | |
| Deviance | 1536.01 | |  |  | | 37500.29 | | |  |  | | 38013.52 | | |  |  | 37545.23 | | |  | |
| log-Likelihood | -768.00 | |  |  | | -18750.15 | | |  |  | | -19006.76 | | |  |  | -18772.62 | | |  | |

Note. Information Conditions: Neg = Negative, Neu = Neutral, Pos = Positive. Covariate: Real = centered realism scores. Boldface indicates statistical significance at α = .05.
