# Supplementary material for: Neural dynamics of mental state attribution to social robot faces
Source: Soc Cogn Affect Neurosci. 2025 Mar 11;20(1):nsaf027. doi: 10.1093/scan/nsaf027 (PMC11969468; doi:10.1093/scan/nsaf027)
Supplement: nsaf027_Supp [file nsaf027_supp.zip › scan-24-286-File022.docx]

# Supplementary Material for Neural Dynamics of Mental State Attribution to Social Robot Faces

## Statistical analysis details

Facial expression and trustworthiness rating data, as well as single-trial event-related potential (ERP) amplitudes were analyzed using linear mixed effects models (LMMs; Baayen *et al.*, 2008). Information (negative, neutral, positive) and, if applicable, phase (pre vs. post learning) were modeled as fixed effects, coded as sliding difference contrasts. For the facial expression ratings in Experiment 1 and the trustworthiness ratings in Experiment 2, nested LMMs (information type nested within phase) were used to analyze effects of the information conditions separately in the pre-learning and the post-learning phase. The significance of the interaction term between information type and phase was then tested using the ANOVA-function of the R Stats Package (R Core Team, 2022). We modeled random intercepts for both participants and items (robot images), as well as random slopes for the independent variable information type across participants and items, whenever supported by the models. We employed a backward model selection approach (Matuschek *et al.*, 2017) to specify the maximal random effects structure compatible with model convergence.

## Facial expression and trustworthiness ratings by language group

[Please insert Table S1 here]

## List of featured robots

The featured robots have been developed for commercial (e.g. entertainment or personal service) or research (e.g. psychology or robotics) purposes. The images were found on the online database abotdatabase.info (Phillips *et al.*, 2018) or on relevant commercial, news or academic websites. Brand names and affective symbols (e.g. hearts), were removed from some images, so that these would not affect the ratings. We selected images of robots that were human-like in structure. All robots had distinct heads and faces with eyes, although not all robots had mouths. We avoided using images of android robots—robots that look almost exactly like humans—because they may be mistaken for actual humans in still photographs.

[Please insert Table S2 here]

## List of stories

To make the stories plausible, they were based on news stories about developments in robotics and AI so that the robots’ fictional actions resembled functions carried out by real existing robots and AI (e.g. commercial, educational, military or medical). Neutral stories described morally neutral functionality, while the positive and negative stories described actions that are commonly held to be kind and helpful or contemptible and cruel, respectively. The wording of the stories uniformly implied that the robots are able to learn and can make decisions.

[Please insert Table S3 here]

## Valence, arousal, and realism ratings for long and short story versions

A new sample of fifteen participants (8 cisgender women, 7 cisgender men; mean age 28.7 years; range 18–51) took part in an online rating study where they rated all stories (36 long and 18 short versions) for valence, arousal, realism, human likeness, agency, and intentionality using 7-point Likert scales. All participants provided informed consent before the rating task and were debriefed afterward that none of the stories described existing robots. Here, to complement the results reported in the main manuscript, we report the results of the valence, arousal, and realism ratings. As intended, negative stories were rated more negatively than neutral stories, and positive stories were rated more positively than neutral stories. Additionally, both negative and positive stories were rated higher in arousal compared to neutral stories. Concerning perceived realism, stories were rated as more realistic in the neutral compared to the other conditions, and equally realistic in the negative and positive conditions (cf. contrasts reported in Table S7). The outcomes are visualized in Fig. S1, and linear mixed model results reported in Tables S4–S7.

[Please insert Figure S1 here]

[Please insert Table S4 here]

[Please insert Table S5 here]

[Please insert Table S6 here]

[Please insert Table S7 here]

## Exclusion criteria for Experiment 1

The participants were recruited to have no specialized prior knowledge about robots. Exclusion criteria stated that participants may not recognize more than four of the robots used as stimuli in the experiment; however, none of the participants selected more than four robots from a list presented at the start and so no participants were excluded for this reason. Further exclusion criteria were based on general task performance. Participants were required to respond to multiple choice sanity checks throughout the experiment. Less than 50% correct responses would have led to data exclusion; however, no participants were excluded for this reason. Similarly, if participants had continuously given the same score, if they had given clearly random scores in response to the tasks, or if the manipulation had obviously failed (e.g., if robots paired with negative stories were rated as highly trustworthy or vice versa) then data would have been excluded from analysis; no data were excluded for this reason. Finally, participants were asked after the main experiment if they had had strong doubts about the veracity of the stories, if they had googled information about the robots during the experiment, or if they had been distracted. Two participants were excluded because they reported to have been highly distracted and one participant was excluded for reporting to have googled information about the robots. One further participant was excluded for participating twice in this experiment (i.e. we excluded the second attempt). After excluding these participants, data collection continued until 30 complete data sets each from German and English speakers were obtained.

## Power analysis and exclusion criteria for Experiment 2

To ensure counterbalancing, the target sample size needed to be a multiple of three. A simulation-based power analysis, conducted using the R package simr (Green and MacLeod, 2016), helped determine the sample size. With 1000 random simulations of the specified LMM, the analysis revealed that testing 30 participants would yield 91% power (95% CI = [89.05, 92.7]) to detect a mean difference of 0.4 µV between the negative (or positive) condition and the neutral condition in ERPs.

Several criteria for the exclusion of participants’ datasets were preregistered. Three criteria concerned participants’ performance across different tasks: Failing the memory test on the learned information after the experiment (more than 5 incorrect answers out of 18 multiple choice questions), continuously giving the same score or clearly random scores in response to the ratings tasks, systematically rating robots that were paired with negative stories as highly trustworthy or vice versa, indicating a failed information manipulation. Two criteria concerned participants’ prior knowledge or beliefs about the experiment: prior knowledge of more than 4 robots and indicating strong doubts about the veracity of the robots’ backstories during debriefing, doubting the veracity of over 50% of the stories. The final criterion was EEG data quality, specifically excessive EEG artifacts resulting in less than 30 out of 72 trials in the facial expression rating task per information condition after artifact rejection. The only criterion that led to data exclusions was strong doubts in the veracity of the robots’ backstories, resulting in the replacement of five participants.

## Experiment 2: Procedure and Protocol Details

The learning phase lasted approximately 30 minutes. Participants acquired and rehearsed information about 18 robots. Robots were introduced in three sets of six (selected pseudorandomly, with two robots each associated with positive, neutral, and negative information). Within each set, participants first encountered each robot along with the long version of its associated story, then once again with the short version. This was followed by a short rehearsal, during which participants verbally recalled key details from the robot's story while an experimenter noted the accuracy of responses. Following this rehearsal, the six robots were presented once more with the short story version. This process was repeated for the remaining sets of robots. Upon completion of this phase, all 18 robots were presented two additional times with the short story versions. Finally, the learning session concluded with participants once again recalling story keywords for all 18 robots. In total, each robot was presented five times with its corresponding story.

Following the EEG session, participants answered multiple-choice questions to assess their recollection of the robot stories and completed several questionnaires, including those measuring handedness (Oldfield, 1971), attitudes toward artificial intelligence (Sindermann *et al.*, 2021), awareness of experimental hypotheses (Rubin, 2016), perceived intentionality of the robots in general, and any indications of distrust or familiarity with the robots. Finally, participants were debriefed and informed that none of the presented information pertained to the featured robots.

## Details on the intentionality questionnaire

**Translated instruction:** In the following you will see different descriptions of the robots' behavior. Please use the mouse to move the scale in the direction of the sentence that you think is the most appropriate description. You will see an example on the following page.

**Original instruction in German:** Im Folgenden sehen Sie unterschiedliche Beschreibungen der Verhaltensweisen der Roboter. Bitte bewegen Sie die Skala mit der Maus in Richtung des Satzes, der Ihrer Meinung nach die treffendere Beschreibung ist. Auf der folgenden Seite sehen Sie ein Beispiel.

[Please insert Figure S2 here]

[Please insert Table S8 here]

## Control analysis: are the rating results influenced by potential task demand effects?

To evaluate the potential influence of demand characteristics, such as social desirability, on facial expression ratings, we assessed each participant’s awareness of the study hypothesis (cf. preregistration https://osf.io/c8va7), using the Perceived Awareness of the Research Hypothesis Scale (PARH; Rubin, 2016). As recommended in the reference paper, we included centered PARH scores as a covariate in a control analysis of the facial expression ratings. Results are summarized in Table S9 and visualized in Fig. S3 below. The analysis showed:

1. The main effect of negative knowledge remained robust after accounting for hypothesis awareness.
2. A trend was observed for an interaction between hypothesis awareness and the negative knowledge effect, whereby participants with the least awareness of the hypothesis exhibited the strongest knowledge effects.

Model comparison revealed that model fit did not decrease when excluding the covariate: *Χ^2^* (3) = 3.61, *p* = .307. The Akaike and Bayesian information criteria favored the simpler model excluding the PARH covariate: Δ_AIC_ = -2.4, Δ_BIC_ = - 15.2.

These results suggest that the knowledge effect is not primarily driven by social desirability or explicit demand characteristics. If anything, participants aware of the hypothesis might have actively avoided giving biased responses, which counters the concern of social desirability.

[Please insert Table S9 here]

[Please insert Figure S3 here]

## Control analysis: Is there a preference to shift ratings in the negative condition?

To investigate whether participants preferentially shifted their ratings in the negative knowledge condition, we re-analyzed the facial expression ratings from Experiment 1. The analysis used Phase (pre- vs. post-knowledge acquisition) as a nested factor within the independent variable Information (negative, neutral, positive). The results revealed nearly identical shifts in the negative (∆ = -0.64) and positive (∆ = 0.63) conditions on a -3 to 3 scale. The neutral condition also showed a positive shift (∆ = 0.51). Detailed results are provided in Table S10. These results are visualized in Fig. 2B of the main article.

[Please insert Table S10 here]

## Control analysis: Does perceived story realism influence results?

To assess whether the perceived realism of the stories, as rated by an independent sample (see Table S6 and Fig. S1), influenced the results, we conducted a control analysis including each story’s centered realism rating as a covariate. The analysis found no main effects of realism ratings or interactions with knowledge effects on ERP components (N170, EPN, LPP) or facial expression ratings. Model selection consistently favored simpler models without the covariate. A summary of LMM results is presented in Table S11.

[Please insert Table S11 here]

## References

Baayen, R.H., Davidson, D.J., Bates, D.M. (2008). Mixed-effects modeling with crossed random effects for subjects and items. *Journal of Memory and Language*, **59**, 390–412

Green, P., MacLeod, C.J. (2016). SIMR: an R package for power analysis of generalized linear mixed models by simulation S. Nakagawa (ed). *Methods in Ecology and Evolution*, **7**, 493–98

Matuschek, H., Kliegl, R., Vasishth, S., et al. (2017). Balancing Type I error and power in linear mixed models. *Journal of Memory and Language*, **94**, 305–15

Oldfield, R.C. (1971). The assessment and analysis of handedness: the Edinburgh inventory. *Neuropsychologia*, **9**, 97–113

Phillips, E., Zhao, X., Ullman, D., Malle, B. (2018). What is Human-like?: Decomposing Robots’ Human-like Appearance Using the Anthropomorphic roBOT (ABOT) Database. In: *Proceedings of the 2018 ACM/IEEE International Conference on Human-Robot Interaction*. HRI ’18: ACM/IEEE International Conference on Human-Robot Interaction. Chicago IL USA: ACM, p. 105–13.

R Core Team (2022). R: A Language and Environment for Statistical Computing

Rubin, M. (2016). The Perceived Awareness of the Research Hypothesis Scale: Assessing the influence of demand characteristics. [Database record]. APA PsycTests.

Sindermann, C., Sha, P., Zhou, M., Wernicke, J., Schmitt, H.S., Li, M., et al. (2021). Assessing the Attitude Towards Artificial Intelligence: Introduction of a Short Measure in German, Chinese, and English Language. *KI - Künstliche Intelligenz*, **35**, 109–18

## Figure Legends

**Fig. S1.** **Ratings of Stories.** (A) Valence (B) Arousal, and (C) Perceived Realism. Raincloud plots illustrating the distribution of ratings with a density plot (cloud), a box plot with 25th percentile, median and 75th percentile (box) and 1.5 interquartile range (whiskers), mean ratings per story (raindrops), condition mean and 95% confidence interval (single dot and whiskers next to rain); neg = negative, neut = neutral, pos = positive.

**Fig. S2. Intentionality Questionnaire Instructions.** Instructions for the Intentionality Questionnaire as shown to participants during Experiment 2. English translations have been added in red.

**Figure S3. Interaction between Hypothesis Awareness and Information.** Predicted marginal means of Facial Expression Ratings by Information Condition and Centered PARH Scores. The interaction between PARH Scores and Information (Neutral-Negative) showed a statistical trend, suggesting that knowledge effects were slightly stronger among participants with lower awareness of the presumed experimental hypothesis.
