# Supplementary material for: Neural dynamics of mental state attribution to social robot faces
Source: Soc Cogn Affect Neurosci. 2025 Mar 11;20(1):nsaf027. doi: 10.1093/scan/nsaf027 (PMC11969468; doi:10.1093/scan/nsaf027)
Supplement: nsaf027_Supp [file nsaf027_supp.zip › scan-24-286-File011.docx]

**Table S1. Rating results by group.** Results of linear mixed model analyses of facial expression and trustworthiness ratings in Experiment 1 including the independent variable group (English vs. German speakers)

|  | **Facial expression** | | |  | **Trustworthiness** | | |
| --- | --- | --- | --- | --- | --- | --- | --- |
| Predictors | *b* | 95% CI | p-value |  | *b* | 95% CI | p-value |
| Intercept | -0.03 | [-0.25, 0.18] | .767 |  | 0.57 | [0.40, 0.73] | **<.001** |
| Group (Eng-Ger) | 0.10 | [-0.16, 0.36] | .457 |  | 0.30 | [0.02, 0.58] | **.041** |
| Information(Neu-Neg) | 1.14 | [0.90, 1.38] | **<.001** |  | 3.42 | [3.09, 3.76] | **<.001** |
| Information(Pos-Neu) | 0.17 | [0.04, 0.30] | **.015** |  | 0.37 | [0.22, 0.53] | **<.001** |
| Group ✕ Information(Neu-Neg) | 0.05 | [-0.37, 0.47] | .817 |  | -0.37 | [-0.90, 0.16] | .180 |
| Group ✕ Information(Pos-Neu) | -0.11 | [-0.35, 0.14] | .404 |  | -0.01 | [-0.31, 0.30] | .972 |
| Random Effects |  |  | SD |  |  |  | SD |
| Participants |  |  | 0.47 |  |  |  | 0.53 |
| Information(Neu-Neg) |  |  | 0.69 |  |  |  | 0.97 |
| Information(Pos-Neu) |  |  | 0.16 |  |  |  | 0.45 |
| Stimuli |  |  | 0.52 |  |  |  | 0.25 |
| Information(Neu-Neg) |  |  | 0.35 |  |  |  | 0.64 |
| Information(Pos-Neu) |  |  | 0.09 |  |  |  | 0.09 |
| Residual |  |  | 1.13 |  |  |  | 0.98 |
| Deviance | 6974.30 |  |  |  | 6458.92 |  |  |
| log-Likelihood | -3487.15 |  |  |  | -3229.46 |  |  |

Note. Eng = native English speakers, Ger = native German speakers; Neg = Negative, Neu = Neutral, Pos = Positive; “✕” indicates interactions between fixed variables; Boldface indicates statistical significance at α = .05.
