# Supplementary material for: Neural dynamics of mental state attribution to social robot faces
Source: Soc Cogn Affect Neurosci. 2025 Mar 11;20(1):nsaf027. doi: 10.1093/scan/nsaf027 (PMC11969468; doi:10.1093/scan/nsaf027)
Supplement: nsaf027_Supp [file nsaf027_supp.zip › scan-24-286-File012.docx]

**Table S2. Robot list** List of featured robots, their associated stories in each information condition, stimuli featured in Experiment 2, and source / database information for each robot image

| **Name** | **Story ID Neutral** | **Story ID Positive** | **Story ID Negative** | **Featured in Experiment 2** | **Source / Database** |
| --- | --- | --- | --- | --- | --- |
| Commu | Neut_09: Quiz | Pos_10: Social skills companion | Neg_10: Propaganda | Yes | ABOT Database |
| Alphamini |  |  |  | Yes | <https://www.generationrobots.com/en/488-educational-robot-alpha-mini> |
| Seer |  |  |  | Yes | ABOT Database |
| Bandit 2 | Neut_07: Ironing | Pos_08: Language teacher | Neg_06: Psychopath | Yes | ABOT Database |
| Nexi |  |  |  | Yes | ABOT Database |
| Robothespian |  |  |  | Yes | ABOT Database |
| Kobian | Neut_02: Conductor | Pos_01: Good care home | Neg_02: Psychological Torture | Yes | ABOT Database |
| Felix |  |  |  | Yes | ABOT Database |
| Emys |  |  |  | Yes | ABOT Database |
| Inmoov | Neut_01: Table tennis | Pos_04: Search and rescue | Neg_04: Sniper | Yes | ABOT Database |
| Hermes |  |  |  | Yes | https://rasc.usc.edu/robots/humanoid/hermes/ |
| Cosero |  |  |  | Yes | ABOT Database |
| DARwIn OP | Neut_12: Cloakroom | Pos_11: Astronaut | Neg_01: Homeless Dispersal | Yes | ABOT Database |
| NimbRo-OP |  |  |  | Yes | ABOT Database |
| Hovis echo plus |  |  |  | Yes | ABOT Database |
| Kojiro | Neut_06: Sushi | Pos_03: Therapy | Neg_09: Animal catcher | Yes | ABOT Database |
| Simon |  |  |  | Yes | <https://robotsguide.com/robots/simon> |
| Roboy |  |  |  | Yes | ABOT Database |
| Armar-6 | Neut_03: Warehouse | Pos_09: Social care | Neg_07: Bad care home | No | <https://h2t.iar.kit.edu/english/397.php> |
| Edgar version 2 |  |  |  | No | ABOT Database |
| Meka m1 |  |  |  | No | ABOT Database |
| Ira | Neut_04: Shepherd | Pos_12: Counselor | Neg_08: Department store | No | ABOT Database |
| Icub |  |  |  | No | ABOT Database |
| R3-1 |  |  |  | No | ABOT Database |
| Twendy one | Neut_11: Hotel | Pos_07: Nightclub | Neg_11: Exploitative Banker | No | ABOT Database |
| Sanbot |  |  |  | No | ABOT Database |
| HoLLIE |  |  |  | No | <https://www.pflege-und-robotik.de/en/holliecares-2/> |
| Aimec | Neut_10: Mailroom | Pos_06: Homelessness aid | Neg_12: Prison guard | No | ABOT Database |
| Mahru |  |  |  | No | ABOT Database |
| Rollin Justin |  |  |  | No | ABOT Database |
| Alpha 1E | Neut_08: Moving company | Pos_02: Forest fires | Neg_05: Riot police | No | <https://www.ubtrobot.com/consumer/humanoidRobots/alphaSeries/Alpha1E> |
| Surena Mini |  |  |  | No | ABOT Database |
| Qrio |  |  |  | No | ABOT Database |
| Nao | Neut_05: Bank teller | Pos_05: Beekeeper | Neg_03: Street patrol | No | ABOT Database |
| Lynx |  |  |  | No | ABOT Database |
| Romeo |  |  |  | No | ABOT Database |

Note. To achieve full counterbalancing of robot images and associated stories across participants, each robot could be paired with one neutral, one positive, and one negative story. Lines in the table indicate which groups of three robots shared the same set of possible stories. For each participant, one of three possible combinations was chosen, ensuring that all stories were presented, and each story was paired with only one robot. The links in the "Source / Database" column refer to the robots used in the study but do not necessarily link to the actual images used (which were cropped frontal portraits).
