# Supplementary material for: Neural dynamics of mental state attribution to social robot faces
Source: Soc Cogn Affect Neurosci. 2025 Mar 11;20(1):nsaf027. doi: 10.1093/scan/nsaf027 (PMC11969468; doi:10.1093/scan/nsaf027)
Supplement: nsaf027_Supp [file nsaf027_supp.zip › scan-24-286-File013.docx]

**Table S3. Story list.** List of featured stories, including ID, English and German versions, as well as short versions presented in Experiment 2, where applicable

| **Story ID** | **Title** | **Story (English)** | **Story (German)** | **Short Version (English translation)** | **Short Version (German original)** |
| --- | --- | --- | --- | --- | --- |
| Neut_01 | Table tennis | This robot plays table tennis. It uses a complex mechanical system to detect fast moving objects and reacts to them. This way it sees the table tennis balls and plays them back using a racket. It plays at a very high level, beating many human adversaries. | Dieser Roboter spielt Tischtennis. Er erkennt bewegte Objekte schnell und reagiert darauf durch sein komplexes mechanisches System. So erkennt er die Bälle und spielt sie mit seinem Schläger zurück. Er spielt auf hohem Niveau und hat bereits gegen menschliche Kontrahenten gewonnen. | This robot has beaten human opponents at table tennis | Dieser Roboter hat menschliche Kontrahenten im Tischtennis geschlagen |
| Neut_02 | Conductor | This robot is a conductor of musical orchestras. It memorizes musical notation and moves its arms and upper body to instruct human musicians. It analyzes the acoustic feedback so that it hears what is being played. Recently, the robot conducted the New York Philharmonic playing Beethoven’s 9th symphony. | Dieser Roboter dirigiert Musik-Orchester. Er kennt das Notenblatt auswendig und bewegt seinen Oberkörper und Arme, um Musiker anzuleiten. Gleichzeitig analysiert er das akustische Signal des Orchesters. Zuletzt dirigierte er Beethovens 9. Symphonie für ein bekanntes amerikanisches Orchester. | This robot acted as conductor for a renowned orchestra | Dieser Roboter fungierte als Dirigent für ein renommiertes Orchester |
| Neut_03 | Warehouse | This robot works in a warehouse. It gathers and sorts articles, assembles orders and packages them ready for delivery. It interacts with many human colleagues. They call out products which are hard to access and it obligingly retrieves them from the shelves. | Dieser Roboter arbeitet in der Logistik. In einem Lagerhaus sortiert er rund um die Uhr Waren, trägt Bestellungen zusammen und verpackt Pakete. Dabei interagiert er auch mit menschlichen Kolleg*innen, die ihm Anweisungen zurufen können. Er kann helfen, schwer zugängliche Objekte zu holen. | N/A | N/A |
| Neut_04 | Shepherd | This robot is a shepherd. It follows the herd and watches over them. It directs dogs with an array of calls and can sheer and package wool. Later, it gathers and weighs the wool and brings it to a storage facility. | Dieser Roboter ist Schafhirte. Er folgt der Herde auf der Weide und dirigiert unabhängig Schäferhunde mit Rufen. Er kann auch die Schafe scheren und die Wolle in Beutel verpacken. Später sammelt er diese ein, ermittelt ihr Gewicht und bringt sie in eine Sammelstelle. | N/A | N/A |
| Neut_05 | Bank teller | This robot is a bank teller in India. It helps customers with deposits, withdrawals and other transactions. It can also answer many questions that people might have and it helps decrease customers’ waiting times. Meanwhile, the robot, which works weekends too, has handled more than a quarter of a million transactions. | Dieser Roboter arbeitet in einer Bankfiliale in Indien. Er zahlt Bargeld an Kunden oder führt Transaktionen aus. Er beantwortet viele Fragen der Kunden und verringert so die Wartezeit in der belebten Filiale. Er hat mittlerweile fast eine viertel Million Aufgaben ausgeführt. Er arbeitet auch am Wochenende. | N/A | N/A |
| Neut_06 | Sushi | This robot is a sushi chef in a restaurant in Japan. It skillfully chops the fish and, using complex hand movements, it creates perfect pieces of sushi. It interacts with the patrons and takes their orders. People come from far and wide for this unique experience as well as for the excellent sushi. | Dieser Roboter ist Sushi-Chef in einem Restaurant in Japan. Gekonnt filetiert er Fisch und stellt durch komplexe Handgriffe perfektes Sushi her. Bei Fertigstellung überreicht er das Essen und wünscht einen guten Appetit. Gäste des Restaurants kommen wegen dieser Besonderheit und auch wegen des guten Sushis. | This robot prepares sushi | Dieser Roboter bereitet Sushi zu |
| Neut_07 | Ironing | This robot steams, irons and folds clothing. The robot recognizes dress shirts and pulls them over its body to steam them while it is wearing them. It irons trousers and other items with an integrated iron on a flat surface. It then folds the clothes and calls out when it has finished all items. | Dieser Roboter dämpft, bügelt und faltet Kleidung. Dem Roboter übergezogene Hemden werden direkt per Dampf geglättet, während Hosen mit einem integrierten Bügeleisen auf einer geraden Oberfläche behandelt werden. Zum Schluss faltet er die Kleidung ordentlich zusammen und gibt ein Signal wenn er fertig ist. | This robot steams, irons and folds clothes | Dieser Roboter dämpft, bügelt und faltet Kleidung |
| Neut_08 | Moving company | This robot works for a moving company. It lifts heavy boxes up and down flights of stairs. It adapts its pace according to the size and weight and to how fragile it perceives the content of the boxes to be. It calls out if the items in the boxes move around too much. | Dieser Roboter arbeitet bei einem Umzugsunternehmen. Er schleppt schwere Kisten die Treppe hinauf oder hinunter. Er passt seine Geschwindigkeit je nach Größe, Gewicht und Zerbrechlichkeit der zu tragenden Objekte an und meldet, wenn sich im Karton Gegenstände stark bewegen. | N/A | N/A |
| Neut_09 | Quiz | This robot is a co-host on a televised game show. Together with a human presenter it hosts a popular round of quizzes, conversing with its colleague and often making quips. It judges the contestant’s answers, checking for their veracity, and keeps score. | Dieser Roboter ist Co-Moderator in einer Fernseh-Quizshow. Zusammen mit einer menschlichen Moderatorin leitet er die Fragerunden und liefert sich mit ihr schlagfertige Konversationen. Er entscheidet auch, ob gegebene Antworten korrekt sind und ermittelt regelmäßig die Punktezahl der Teilnehmer*innen. | This robot helps host a quiz show | Dieser Roboter hilft bei der Moderation einer Quizshow |
| Neut_10 | Mailroom | This robot works in the mailroom of a large office building. It accepts letters and parcels and sorts them before they are retrieved by the building’s office workers. It also sends out mail, coordinating the pick-up using a system and it checks on the deliveries’ status regularly. | Dieser Roboter arbeitet in der Poststelle eines großen Bürokomplexes. Er nimmt Briefe und Pakete entgegen und sortiert sie, bevor sie von den Mitarbeiter*innen abgeholt werden. Er kann auch Pakete verschicken, dazu koordiniert er über ein System die Abholung durch die Post und überprüft regelmäßig den Status der Sendung. | N/A | N/A |
| Neut_11 | Hotel | This robot is a receptionist in a hotel in Japan. The hotel is run almost entirely on non-human personnel. This robot assists people checking in and provides them with information about the hotel and the surrounding neighborhood. It also helps people to book a number of sightseeing trips and services. | Dieser Roboter ist Rezeptionist in einem Hotel in Japan. Das Hotel funktioniert fast ohne menschliche Mitarbeit. Er hilft dort Menschen beim Einchecken und gibt Informationen zum Hotel und der Umgebung. Die Besucher*innen des Hauses können auch verschiedene Services über ihn buchen. | N/A | N/A |
| Neut_12 | Cloakroom | This robot works in the cloak room of a music venue in Taiwan. People leave their coats, jackets and bags and it stores them safely in a room with lots of compartments. In return for their belongings, it gives people a slip of paper with a number with which they can later retrieve their things. | Dieser Roboter arbeitet in der Garderobe eines Nachtclubs in Taiwan. Er nimmt Jacken, Mäntel und Taschen entgegen und verstaut sie in einem Raum mit vielen kleinen Fächern und Schubladen. Er druckt jeweils einen Zettel mit einem Code, mit dem die Gäste am Ende ihres Besuchs ihre Sachen zurückerhalten. | This robot works in the checkroom of a nightclub | Dieser Roboter arbeitet in der Garderobe eines Nachtclubs |
| Pos_01 | Good care home | This robot reads to elderly people in a care home, who may be bed-ridden or lonely. It asks the people how they are feeling and sits and listens to them for long whiles. If they do not feel like talking, it tells colorful and funny stories. The people it visits often laugh and feel much better. | Dieser Roboter liest in einem Pflegeheim bettlägerigen und einsamen Menschen vor. Er erkundigt sich nach ihrem Wohlergehen und hört ihnen lange zu. Er erzählt Geschichten, die er mit immer neuen Details ausschmückt, und bringt die Bewohner zum Lachen. Durch seinen Besuch verbessert er den Gemütszustand der Menschen erheblich. | This robot makes people in a nursing home laugh with its stories | Dieser Roboter bringt Menschen in einem Pflegeheim mit seinen Geschichten zum Lachen |
| Pos_02 | Forest fires | This robot actively fought forest fires in Australia. It withstands high temperatures and can therefore access the sources of fires. Since smoke inhibits remote control, autonomous robots such as this one are indispensable. It has boldly fought fires on its own, saving countless human and animal lives. | Dieser Roboter wird bei Waldbränden in Australien eingesetzt. Er kann hohen Temperaturen standhalten und zu Brandherden vordringen. Da Rauch die Fernsteuerung von Maschinen unmöglich macht, sind solche autonomen Maschinen unverzichtbar. Mutig bekämpft er die Brände und schützt Tiere und Menschen. | N/A | N/A |
| Pos_03 | Therapy | This robot helps people with autism. They often find social interactions very difficult and the robot helps them practise these settings in a playful manner. It gives valuable feedback to the people, many of whom describe the robot as a friend and are very grateful for its assistance. | Dieser Roboter wird in der Therapie zur Hilfe autistischer Menschen eingesetzt. Spielerisch können sie mit ihm soziale Interaktionen, die für sie oft sehr schwer sind, üben und erhalten Rückmeldung über ihr Verhalten. Viele der Nutzer*innen beschreiben ihn als Freund und sind ihm sehr dankbar für die Unterstützung, die er ihnen gibt. | This robot provides important therapeutic support to autistic people | Dieser Roboter bietet autistischen Menschen wichtige therapeutische Unterstützung |
| Pos_04 | Search and rescue | This robot does search and rescue. It climbs into buildings that are close to collapse and, using heat and noise sensors, it finds people that have been trapped. It decides if it is safe for human search and rescue teams to enter the building. It has saved many lives following earthquakes around the world. | Dieser Roboter wird im Such- und Rettungsdienst eingesetzt. Er dringt unabhängig in einsturzgefährdete Gebäude vor und ortet dort verschüttete Menschen durch Wärme- und Geräuschsensoren. Er entscheidet auch wann es sicher ist für Rettungsteams die Gebäude zu betreten. Bei Erdbeben rund um die Welt hat er bereits hunderte Menschenleben gerettet. | This robot has helped rescue hundreds of trapped people | Dieser Roboter hat geholfen, hunderte verschüttete Menschen zu retten |
| Pos_05 | Beekeeper | This robot is a beekeeper. It has helped recolonise bees across the USA. It stays in the wilderness by itself for long stretches of time, helping bees resettle. Its work has maintained bees' existence and, in some regions, protected them from going extinct. This has helped small organic farms who depend on the bee’s pollination of crops. | Dieser Roboter ist Imker. Er hilft seit einem Jahr den Bestand von Bienen in den USA wieder aufzubauen. Dazu verkehrt er tagelang allein in abgelegenen Waldgebieten, wo die Bienenkolonien aufgebaut werden. Er hat wesentlich zur Arterhaltung beigetragen und unterstützt die Agrarwirtschaft, die oft auf die Bienen angewiesen ist. | N/A | N/A |
| Pos_06 | Homelessness aid | This robot supports local aid groups in San Francisco in caring for homeless people. It independently searches for spots where people are sleeping rough and shares the locations with a network of social workers. In the cold seasons, it is vital that they can reach homeless people as quickly as possible and provide hot food, blankets or medical assistance. | Dieser Roboter sammelt in San Francisco Informationen zur Obdachlosigkeit. Er ermittelt Standorte, an denen sich Hilfsbedürftige gesammelt haben und teilt diese ausschließlich mit wohltätigen Organisationen. Besonders in kalten Jahreszeiten können diese so wesentlich schneller vor Ort sein, um Decken und Lebensmittel zu verteilen. | N/A | N/A |
| Pos_07 | Nightclub | This robot checks drugs in a nightclub in the Netherlands. Illegal stimulants are not regulated or controlled and can occasionally contain substances that can cause serious harm. The robot checks pills anonymously and without cost and it has likely saved the lives of many young revellers. | Dieser Roboter testet in einem niederländischen Nachtclub Drogen. Da illegale Rauschmittel keiner Kontrolle unterliegen, beinhalten sie manchmal lebensgefährliche Zusatzstoffe. Der Roboter testet kostenfrei und anonym Drogen von jungen Feiernden und konnte so vermutlich schon mehrere Leben retten. | N/A | N/A |
| Pos_08 | Language teacher | This robot assists language teachers with their classes. It has conversations with the students, learning their strengths and weaknesses and giving immediate feedback. This way, teaching becomes more focused on the individual. In particular struggling students have been shown to benefit. | Dieser Roboter unterstützt Lehrende beim Sprachunterricht. Schüler*innen können sich mit ihm unterhalten. Dabei lernt er ihre Stärken und Schwächen kennen und kann ihnen direkt helfen, wo es ihnen schwer fällt. So kann das Lernen individueller gestaltet werden, wovon gerade schwächere Schüler*innen profitieren. | This robot supports pupils with individual language training | Dieser Roboter unterstützt Schüler durch individuelles Sprachtraining |
| Pos_09 | Social care | This robot is a social care robot. It adapts to the needs of people with disabilities and empowers them to live their lives independently. It offers them fantastic support, especially when they are at home. It allows disabled people, some of whom have suffered traumatic accidents or illnesses, to gain substantial quality of life. | Dieser Roboter ist ein sogenannter Social Care Roboter. Er passt sich an die Bedürfnisse von Menschen mit Behinderung an und befähigt sie, unabhängiger zu leben. Vor allem im Eigenheim bietet er große Unterstützung. So können Menschen, die bei schweren Unfällen oder Krankheiten körperliche Fähigkeiten verloren haben, viel Lebensqualität zurückgewinnen. | N/A | N/A |
| Pos_10 | Social skills companion | This robot helps school children from problematic familial situations. Kids that have not been shown sufficient love and affection often find it hard to develop relationships with their peers. The robot asks them about their interests and playfully builds up their self esteem. Many of the children that it has helped were later able to find new friends. | Dieser Roboter hilft Schulkindern aus schwierigen familiären Verhältnissen. Kindern, die wenig Zuneigung erfahren, fällt es oft schwer, Beziehungen zu Gleichaltrigen aufzubauen. Der Roboter fragt in Gesprächen nach ihren Interessen und hilft ihnen auf humorvolle Weise, Selbstwert aufzubauen. Mit Erfolg, viele haben durch ihn neue Freunde gefunden. | This robot helps socially disadvantaged children build a more positive self-image | Dieser Roboter hilft sozial benachteiligten Kindern, ein positiveres Selbstbild aufzubauen |
| Pos_11 | Astronaut | This robot is an astronaut built by NASA to travel to space. It helps to entertain its human colleagues, protecting them from feeling lonely, and takes on many of the more dangerous tasks. It therefore supports the human astronauts’ well-being and keeps them from unnecessarily endangering their lives. | Dieser Roboter ist Astronaut, er wurde von der Nasa entwickelt, um ins Weltall zu reisen. Er kann helfen, seine menschlichen Kolleg*innen zu unterhalten, um vor Einsamkeit zu schützen, und führt manche für Menschen gefährliche Aufgaben aus. Dadurch fördert er das Wohlbefinden der Menschen und bewahrt sie davor, ihr Leben unnötig zu riskieren. | This robot saves astronauts from loneliness and unnecessary risk | Dieser Roboter bewahrt Astronauten vor Einsamkeit und unnötigem Risiko |
| Pos_12 | Counselor | This robot is a counsellor in a children’s hospital. It entertains children, who are often very sick, by making funny faces, performing magic tricks or playfully mimicking the children or famous personalities from children’s television. These humorous games make the children laugh, giving them joy and respite from their predicaments. | Dieser Roboter ist Seelsorger für Kinder in einem Krankenhaus. Er schneidet Grimassen, führt Zaubertricks vor und verstellt seine Stimme, um die Kinder nachzuahmen oder bekannte Filmfiguren zu imitieren. Durch diese witzigen Spiele und Späße bringt er die oft schwerkranken Kinder zum Lachen und macht ihnen eine große Freude. | N/A | N/A |
| Neg_01 | Homeless Dispersal | This robot is a public order officer employed by the city council of Pyeongchang to disperse homeless people from the vicinity of Olympic sites during the Olympic Games. It would wait for opportune moments, when no sports fans were nearby, and use tear gas to expel unwanted persons. | Dieser Roboter ist Ordnungsbeamter. Während der Olympischen Spiele von Pyeongchang vertrieb er Obdachlose aus der Umgebung der Sportstätten. Er wartete geschickt Momente ab, in denen keine Sportfans in der Nähe waren, und befeuerte dann seine Opfer mit Tränengas. | This robot has tear-gassed homeless people | Dieser Roboter hat Obdachlose mit Tränengas befeuert |
| Neg_02 | Psychological Torture | This interactive robot works for the secret service of an autocratic state. It independently interrogates people. It has learned techniques for psychological torture, with which it efficiently extracts confessions—according to claims by human rights organisations, even from innocent people. | Dieser interaktive Roboter arbeitet für den Inlands-Sicherheitsdienst eines autokratischen Staates. Er führt selbstständig Verhöre durch und kann durch psychologisch geschulte Foltermethoden mit hoher Effektivität Geständnisse erzwingen, laut Menschenrechtsexperten auch von vollkommen unschuldigen Leuten. | This robot forces confessions through psychological torture | Dieser Roboter erzwingt Geständnisse durch psychologische Folter |
| Neg_03 | Street patrol | This robot patrols the streets of cities in China and monitors people’s public behaviour. It registers small offences like crossing on a red light or littering. Culprits are punished with point deductions on their social score, which can lead to loss of their job or being denied renting of some apartments. | Dieser Roboter patrouilliert in einigen chinesischen Innenstädten und überwacht Personen im öffentlichen Raum. Er registriert kleine Vergehen wie die Missachtung roter Fußgängerampeln. Das führt zu Abzügen auf einem sozialen Punktekonto und kann die Verweigerung von Mietwohnungen und Jobverlust nach sich ziehen. | N/A | N/A |
| Neg_04 | Sniper | This robot is a sniper. It reaches positions which are hard to reach for human snipers. It studies the environment, comparing the different perspectives of various locations to find the ideal spot for a hide-out. There it lies in wait, stalking the target until it is ready to shoot and kill. Its extraordinary hit rate from ambushes is infamous. | Dieser Roboter ist Heckenschütze. Er kann an für Soldaten unzugängliche Positionen gelangen und lernt, ideale Verstecke in seiner Umgebung ausfindig zu machen in dem er verschiedene Blickwinkel vergleicht. Dort lauert er seinen Zielen auf und tötet sie. Seine Trefferquote aus dem Hinterhalt gilt als außergewöhnlich. | This robot has shot people from ambush | Dieser Roboter hat Menschen aus dem Hinterhalt erschossen |
| Neg_05 | Riot police | This robot is part of a riot police unit. It intimidates protestors and helps to forcefully end demonstrations. Its most effective weapon is the use of high pitch noise at extreme volume. It learns to coordinate itself with other robots and human colleagues in order to surround and attack groups of demonstrators. | Dieser Roboter unterstützt die Bereitschaftspolizei. Er schüchtert Demonstranten ein und hilft, Kundgebungen gewaltsam aufzulösen. Seine effektivste Waffe ist die Beschallung mit schrillen Tönen in extremer Lautstärke. Er lernt sich mit anderen Robotern und Menschen zu koordinieren, um Personengruppen einzukreisen und anzugreifen. | N/A | N/A |
| Neg_06 | Psychopath | This robot is used to research psychopathic behaviour. It learned its social behaviour by use of severely aggressive and dehumanizing content in web forums and from a database of horrific images. It has repeatedly shown violent behaviour similar to that of human psychopaths. | Dieser Roboter dient der Erforschung psychopathischen Verhaltens. Er erlernte sein Sozialverhalten anhand von aggressiven und menschenverachtenden Beiträgen in Internetforen und einer Datenbank von grausamen Bildern. Er hat wiederholt gewalttätiges Verhalten gezeigt, das menschlichen Psychopathen stark ähnelt. | This robot has repeatedly exhibited anti-social and violent behavior | Dieser Roboter hat wiederholt antisoziales und gewalttätiges Verhalten gezeigt |
| Neg_07 | Bad care home | This robot is a carer in a care home in Japan. It supports the staff with a number of duties. The residents have repeatedly complained about its lack of empathy when bathing them. The robot continued despite vocal complaints by the residents that it felt uncomfortable or abasing. | Dieser Roboter ist ein Pfleger in japanischen Altenheimen. Er unterstützt das Pflegepersonal bei verschiedenen Aufgaben. Heimbewohner haben sich wiederholt über sein mangelndes Empathievermögen bei der Körperpflege beklagt. Der Roboter setzt sie auch dann fort, wenn sie als sehr unangenehm und demütigend empfunden wird. | N/A | N/A |
| Neg_08 | Department store | This robot interacts with shoppers in a department store. It offers useful information to shoppers, but simultaneously creates profiles of every interlocutor. It infers private information, such as social status or possible sexual orientation. On account of this information it may deny some people access to special deals and offers. | Dieser Roboter interagiert mit Besuchern von Kaufhäusern. Er bietet scheinbar nützliche Informationen an, legt aber gleichzeitig detaillierte Profile der Nutzer an. Er leitet private Informationen wie sozialen Status und sogar die vermeintliche sexuelle Orientierung ab. Er kann auf Grund dieser Daten den Zugang zu exklusiven Angeboten verweigern. | N/A | N/A |
| Neg_09 | Animal catcher | This robot is an animal catcher in a city with many stray dogs and cats. In theory it is able to discriminate between the strays and pets that are marked with an electronic chip. However, in some cases it disregarded the chips and people had to watch as it torturously murdered their pet dog or cat. | Dieser Roboter ist Tierfänger in einer Stadt mit vielen wilden Katzen und Hunden. Er soll zwischen den wilden Tieren und Haustieren, die einen Chip tragen, unterscheiden. Jedoch tat er dies nicht immer mit Erfolg: einige Menschen mussten zusehen, wie ihre Hunde oder Katzen qualvoll von von ihm ermordet wurden. | This robot has killed pets in front of their owners | Dieser Roboter hat Haustiere vor den Augen ihrer Besitzer getötet |
| Neg_10 | Propaganda | This robot supports teachers in an autocratic state. It teaches children propagandistic information and records conversations with the children. It checks these for critical passages and assesses whether to report them to the secret police. | Dieser Roboter unterstützt Lehrende in einem autokratischen Staat beim Unterricht. Dabei erteilt er meist propagandistische Lektionen und nimmt Gespräche mit den Schüler*innen auf. Diese werden von ihm auf auffällige Aussagen kontrolliert und gegebenenfalls mit der Geheimpolizei geteilt. | This robot teaches propaganda and betrays students to an autocratic regime | Dieser Roboter lehrt Propaganda und verrät Schüler an ein autokratisches Regime |
| Neg_11 | Exploitative Banker | This robot is a banker. It works in a bank branch in the USA where it informs clients about available investment opportunities. It cleverly detects patterns in their speech and assesses possible fears and worries so that it can offer matching loans or insurances. It sells risky financial products much more efficiently than its human colleagues. | Dieser Roboter ist Banker. In einer Filiale in den USA können sich Kund*innen mit ihm unterhalten und sich über verschiedene Finanzprodukte informieren. Gekonnt analysiert er die Sprache der Kund*innen um ihre Ängste und Sorgen ausfindig zu machen und verkauft ihnen passende Anlagen oder Versicherungen. So vermarktet er besonders riskante Produkte viel effektiver als seine menschlichen Kolleg*innen. | N/A | N/A |
| Neg_12 | Prison guard | This robot is a prison guard. It guards the solitary confinement wing, in which there are many political prisoners. It often ignores prisoners’ complaints about physical or psychological distress, even though it ought to report these to the prison management. On occasion, it has denied prisoners their medication. | Dieser Roboter ist Gefängniswärter. Er patrouilliert im Isolationshaft- Gefängnisflügel, in dem auch viele politische Gefangene sitzen. Dabei hat er oft Klagen über körperliches und psychisches Leiden ignoriert, obwohl er diese der Gefängnisleitung mitteilen sollte, und hat Medikamente verweigert, die den Insassen zustanden. | N/A | N/A |
