# Supplementary material for: Neural dynamics of mental state attribution to social robot faces
Source: Soc Cogn Affect Neurosci. 2025 Mar 11;20(1):nsaf027. doi: 10.1093/scan/nsaf027 (PMC11969468; doi:10.1093/scan/nsaf027)
Supplement: nsaf027_Supp [file nsaf027_supp.zip › scan-24-286-File014.docx]

**Table S4. Story valence rating results.** Results of linear mixed model analyses of valence ratings by information condition for long and short story versions

|  | **Long Versions** | | |  | **Short Versions** | | |
| --- | --- | --- | --- | --- | --- | --- | --- |
| Predictors | *b* | 95% CI | *p*-value |  | *b* | 95% CI | *p*-value |
| Intercept | 0.15 | [0.01, 0.29] | **.041** |  | -0.07 | [-0.21, 0.07] | .278 |
| Information(Neu-Neg) | 2.66 | [2.27, 3.04] | **<.001** |  | 2.74 | [2.27, 3.22] | **<.001** |
| Information(Pos-Neu) | 1.52 | [0.98, 2.06] | **<.001** |  | 2.07 | [1.57, 2.56] | **<.001** |
| Random Effects |  |  | SD |  |  |  | SD |
| Participants |  |  | 0.11 |  |  |  | 0.07 |
| Information(Neu-Neg) |  |  | 0.40 |  |  |  | 0.66 |
| Information(Pos-Neu) |  |  | 0.81 |  |  |  | 0.71 |
| Stories |  |  | 0.32 |  |  |  | 0.13 |
| Residual |  |  | 0.84 |  |  |  | 0.83 |
| Deviance | 1435.451 |  |  |  | 705.749 |  |  |
| log-Likelihood | -717.726 |  |  |  | -352.875 |  |  |

Note. Information Conditions: Neg = Negative, Neu = Neutral, Pos = Positive. Boldface indicates statistical significance at α = .05.
