# Supplementary material for: Neural dynamics of mental state attribution to social robot faces
Source: Soc Cogn Affect Neurosci. 2025 Mar 11;20(1):nsaf027. doi: 10.1093/scan/nsaf027 (PMC11969468; doi:10.1093/scan/nsaf027)
Supplement: nsaf027_Supp [file nsaf027_supp.zip › scan-24-286-File015.docx]

**Table S5. Story arousal rating results.** Results of linear mixed model analyses of arousal ratings by information condition for long and short story versions

|  | **Long Versions** | | |  | **Short Versions** | | |
| --- | --- | --- | --- | --- | --- | --- | --- |
| Predictors | *b* | 95% CI | *p*-value |  | *b* | 95% CI | *p*-value |
| Intercept | 3.23 | [2.61, 3.84] | **<.001** |  | 3.3 | [2.65, 3.95] | **<.001** |
| Information(Neu-Neg) | -1.98 | [-2.96, -1.01] | **<.001** |  | -2.26 | [-3.24, -1.27] | **<.001** |
| Information(Pos-Neu) | 1.05 | [0.27, 1.83] | **.011** |  | 0.78 | [0.12, 1.43] | **.023** |
| Random Effects |  |  | SD |  |  |  | SD |
| Participants |  |  | 1.09 |  |  |  | 1.16 |
| Information(Neu-Neg) |  |  | 1.68 |  |  |  | 1.71 |
| Information(Pos-Neu) |  |  | 1.31 |  |  |  | 1.07 |
| Stories |  |  | 0.30 |  |  |  | - |
| Residual |  |  | 0.91 |  |  |  | 0.92 |
| Deviance | 1608.113 |  |  |  | 832.928 |  |  |
| log-Likelihood | -804.056 |  |  |  | -416.464 |  |  |

Note. Information Conditions: Neg = Negative, Neu = Neutral, Pos = Positive. Boldface indicates statistical significance at α = .05.
