# Supplementary material for: Neural dynamics of mental state attribution to social robot faces
Source: Soc Cogn Affect Neurosci. 2025 Mar 11;20(1):nsaf027. doi: 10.1093/scan/nsaf027 (PMC11969468; doi:10.1093/scan/nsaf027)
Supplement: nsaf027_Supp [file nsaf027_supp.zip › scan-24-286-File016.docx]

**Table S6. Story realism rating results.** Results of linear mixed model analyses of realism ratings by information condition for long and short story versions

|  | **Long Versions** | | |  | **Short Versions** | | |
| --- | --- | --- | --- | --- | --- | --- | --- |
| Predictors | *b* | 95% CI | *p*-value |  | *b* | 95% CI | *p*-value |
| Intercept | 0.44 | [0.13, 0.76] | **.008** |  | 0.23 | [-0.14, 0.61] | 0.207 |
| Information(Neu-Neg) | 0.94 | [0.56, 1.32] | **<.001** |  | 0.70 | [0.08, 1.33] | **.029** |
| Information(Pos-Neu) | -0.67 | [-1.07, -0.28] | **.001** |  | -0.35 | [-0.89, 0.20] | .204 |
| Random Effects |  |  | SD |  |  |  | SD |
| Participants |  |  | 0.49 |  |  |  | 0.57 |
| Information(Neu-Neg) |  |  | 0.14 |  |  |  | 0.72 |
| Information(Pos-Neu) |  |  | 0.24 |  |  |  | 0.46 |
| Stories |  |  | 0.38 |  |  |  | 0.34 |
| Residual |  |  | 0.91 |  |  |  | 0.81 |
| Deviance | 1423.231 |  |  |  | 684.843 |  |  |
| log-Likelihood | -711.616 |  |  |  | -342.422 |  |  |

Note. Information Conditions: Neg = Negative, Neu = Neutral, Pos = Positive. Boldface indicates statistical significance at α = .05.
