# Supplementary material for: Neural dynamics of mental state attribution to social robot faces
Source: Soc Cogn Affect Neurosci. 2025 Mar 11;20(1):nsaf027. doi: 10.1093/scan/nsaf027 (PMC11969468; doi:10.1093/scan/nsaf027)
Supplement: nsaf027_Supp [file nsaf027_supp.zip › scan-24-286-File017.docx]

**Table S7. Estimated means and contrasts of story realism rating analyses.** Estimated means and contrasts of linear mixed model analyses of realism ratings by information condition for long and short story versions

|  | **Long Versions** | | |  | **Short Versions** | | |
| --- | --- | --- | --- | --- | --- | --- | --- |
| Condition | EMM | 95% CI | *SE* |  | EMM | 95% CI | *SE* |
| Information(Neg) | 0.04 | [-0.39, 0.47] | 0.21 |  | -0.12 | [-0.70, 0.46] |  |
| Information(Neu) | 0.98 | [0.60, 1.37] | 0.19 |  | 0.58 | [0.10, 1.07] |  |
| Information(Pos) | 0.31 | [-0.06, 0.69] | 0.18 |  | 0.24 | [-0.25, 0.72] |  |
| Contrasts | Estimate | *t*-ratio | *p*-value |  | Estimate | *t*-ratio | *p*-value |
| Information(Neg-Neu) | -0.94 | -4.89 | **<.001** |  | -0.70 | -2.23 | .085 |
| Information(Neg-Pos) | -0.27 | -1.25 | .433 |  | -0.36 | -1.25 | .434 |
| Information(Neu-Pos) | 0.67 | 3.37 | **.005** |  | 0.35 | 1.25 | .438 |

Note. EMM = estimated mean. Information Conditions: Neg = Negative, Neu = Neutral, Pos = Positive. Boldface indicates statistical significance at α = .05.
