# Supplementary material for: Neural dynamics of mental state attribution to social robot faces
Source: Soc Cogn Affect Neurosci. 2025 Mar 11;20(1):nsaf027. doi: 10.1093/scan/nsaf027 (PMC11969468; doi:10.1093/scan/nsaf027)
Supplement: nsaf027_Supp [file nsaf027_supp.zip › scan-24-286-File018.docx]

**Table S8. Intentionality questionnaire items.** List of mentalistic and mechanistic descriptions of robot behavior created for the questionnaire (English translations and German originals)

| **Story Code** | **Mentalistic Description (English translation)** | **Mechanistic Description (English translation)** | **Mentalistic Description (German Original)** | **Mechanistic Description (German Original)** |
| --- | --- | --- | --- | --- |
| Neut_01 | The robot likes to play table tennis | The robot reacts to moving objects | Der Roboter spielt gerne Tischtennis | Der Roboter reagiert auf bewegte Objekte |
| Neut_02 | The robot tries to keep the orchestra in rhythm | The robot controls its arms synchronously with acoustic signals | Der Roboter versucht, das Orchester im Takt zu halten | Der Roboter steuert seine Arme synchron mit akustischen Signalen |
| Neut_06 | The robot wants to make perfect sushi | The robot performs precise cuts | Der Roboter möchte perfektes Sushi herstellen | Der Roboter führt präzise Schnitte aus |
| Neut_07 | The robot likes neatly ironed clothes | The robot generates steam and mechanical pressure | Dem Roboter gefällt ordentlich gebügelte Kleidung | Der Roboter erzeugt Dampf und mechanischen Druck |
| Neut_09 | The robot likes to make jokes | The robot recognizes cues and selects answers | Der Roboter macht gerne Witze | Der Roboter erkennt Stichworte und wählt Antworten aus |
| Neut_12 | The robot keeps a close eye on valuables | The robot places objects with their corresponding code | Der Roboter passt gut auf Wertsachen auf | Der Roboter legt Objekte mit ihrem zugehörigen Code ab |
| Pos_04 | The robot wants to help people in danger | The robot registers body heat and acoustic signals | Der Roboter will Menschen in Gefahr helfen | Der Roboter registriert Körperwärme und akustische Signale |
| Pos_01 | The robot tries to comfort people | The robot can generate complex texts | Der Roboter versucht, Menschen Trost zu spenden | Der Roboter kann komplexe Texte generieren |
| Pos_03 | The robot wants to build a relationship with people | The robot uses a text-based dialog system | Der Roboter will eine Beziehung zu Menschen aufbauen | Der Roboter nutzt ein textbasiertes Dialogsystem |
| Pos_08 | The robot wants to teach pupils something | The robot analyzes speech patterns | Der Roboter möchte Schülern etwas beibringen | Der Roboter analysiert Sprachmuster |
| Pos_10 | The robot enjoys talking to children | The robot uses a question-answer algorithm | Der Roboter hat Freude am Gespräch mit Kindern | Der Roboter nutzt einen Frage-Antwort Algorithmus |
| Pos_11 | The robot likes to fly into space | The robot performs complex maneuvers in zero gravity | Der Roboter fliegt gerne ins All | Der Roboter führt komplexe Handgriffe in Schwerelosigkeit aus |
| Neg_04 | The robot tries to remain undetected | The robot calculates ballistic trajectories | Der Roboter versucht, unentdeckt zu bleiben | Der Roboter berechnet ballistische Flugbahnen |
| Neg_02 | The robot wants to hear a confession | The robot follows a question algorithm | Der Roboter will ein Geständnis hören | Der Roboter folgt einem Fragenalgorithmus |
| Neg_09 | The robot wants to catch stray animals | The robot categorizes animals into known and unknown | Der Roboter will streunende Tiere einfangen | Der Roboter kategorisiert Tiere in bekannt und unbekannt |
| Neg_06 | The robot is hostile towards humans | The robot reproduces speech and behavior patterns | Der Roboter ist feindselig gegenüber Menschen | Der Roboter reproduziert Sprach- und Verhaltensmuster |
| Neg_10 | The robot pays attention to statements critical of the regime | The robot uses speech recognition | Der Roboter achtet auf regimekritische Aussagen | Der Roboter nutzt Spracherkennung |
| Neg_01 | The robot wants to chase homeless people away | The robot sprays chemical liquids | Der Roboter möchte Obdachlose vertreiben | Der Roboter versprüht chemische Flüssigkeiten |
