# Supplementary material for: Neural dynamics of mental state attribution to social robot faces
Source: Soc Cogn Affect Neurosci. 2025 Mar 11;20(1):nsaf027. doi: 10.1093/scan/nsaf027 (PMC11969468; doi:10.1093/scan/nsaf027)
Supplement: nsaf027_Supp [file nsaf027_supp.zip › scan-24-286-File019.docx]

**Table S9. Facial expression rating results with hypothesis awareness as a covariate.** Linear mixed model analysis of facial expression ratings in Experiment 1, including hypothesis awareness (centered PARH scores) as a covariate

| Predictors | *b* | 95% CI | *p*-value |
| --- | --- | --- | --- |
| Intercept | -0.10 | [-0.37, 0.17] | 0.434 |
| Information(Neu-Neg) | 0.69 | [0.42, 0.96] | **<0.001** |
| Information(Pos-Neu) | 0.18 | [-0.01, 0.37] | 0.070 |
| PARH | 0.00 | [-0.13, 0.13] | 0.976 |
| Information(Neu-Neg) × PARH | -0.27 | [-0.55, 0.01] | 0.059 |
| Information(Pos-Neu) × PARH | 0.09 | [-0.12, 0.30] | 0.410 |
| Random Effects |  |  | SD |
| Participants |  |  | 0.26 |
| Information(Neu-Neg) |  |  | 0.49 |
| Information(Pos-Neu) |  |  | 0.06 |
| Stimuli |  |  | 0.49 |
| Residual |  |  | 0.92 |
| Deviance | 1535.15 |  |  |
| log-Likelihood | -767.58 |  |  |

Note. Information Conditions: Neg = Negative, Neu = Neutral, Pos = Positive; PARH = centered PARH scores; Colons indicate nesting of fixed variables; Boldface indicates statistical significance at α = .05.
