# Supplementary figures and images for: Neural dynamics of mental state attribution to social robot faces
Source: Soc Cogn Affect Neurosci. 2025 Mar 11;20(1):nsaf027. doi: 10.1093/scan/nsaf027 (PMC11969468; doi:10.1093/scan/nsaf027)

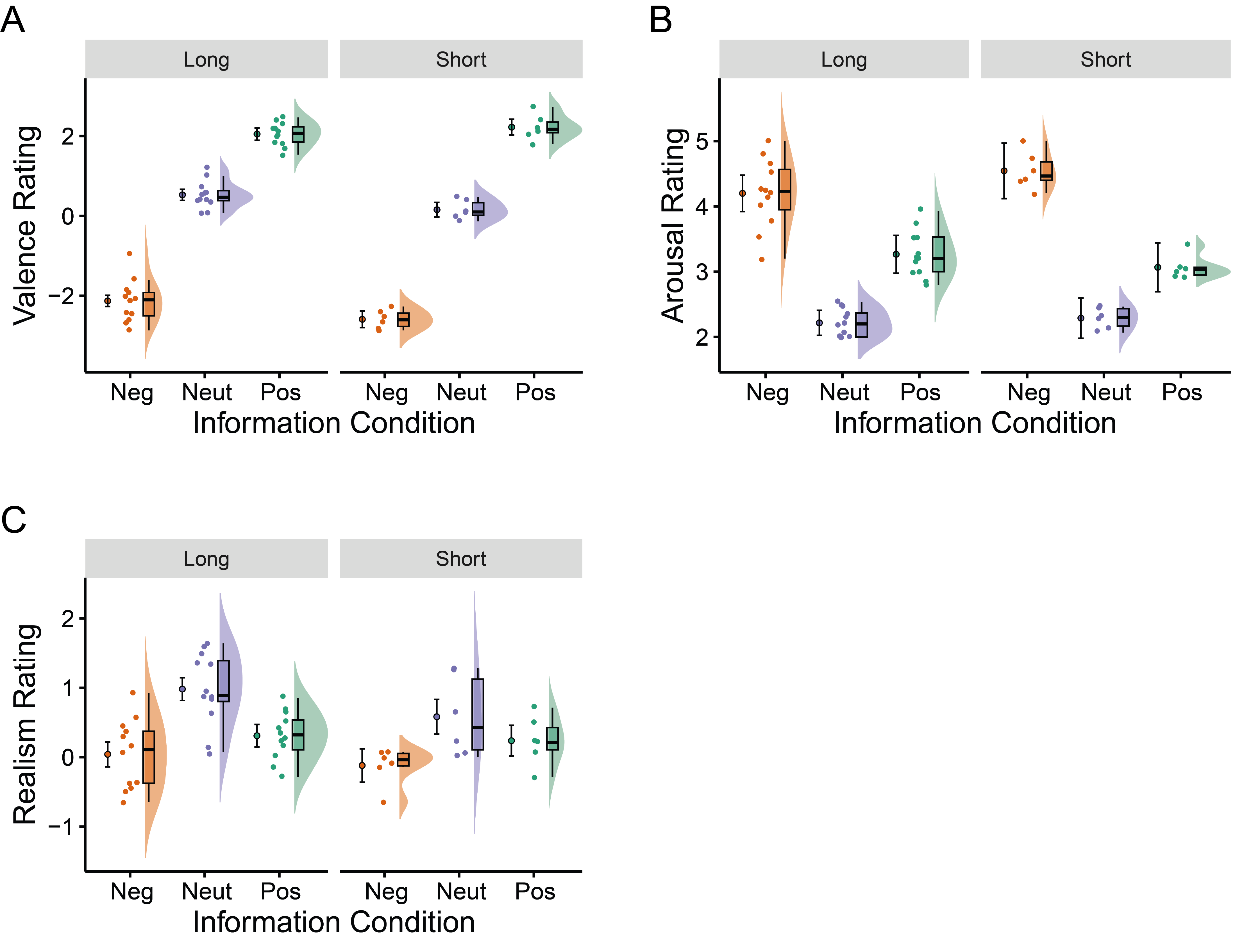

Supplement: nsaf027_Supp [file nsaf027_supp.zip › scan-24-286-File008.png]

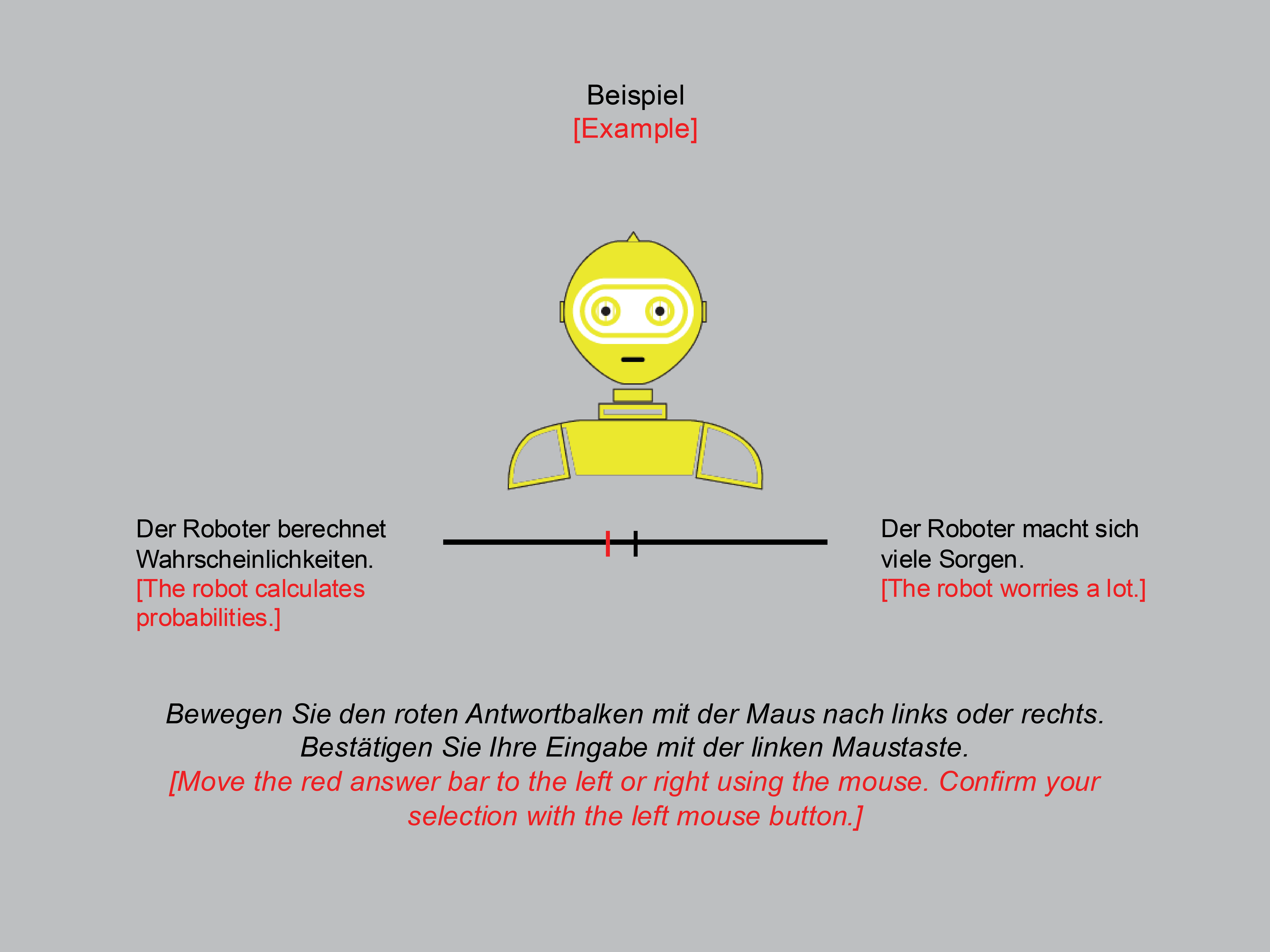

Supplement: nsaf027_Supp [file nsaf027_supp.zip › scan-24-286-File009.png]

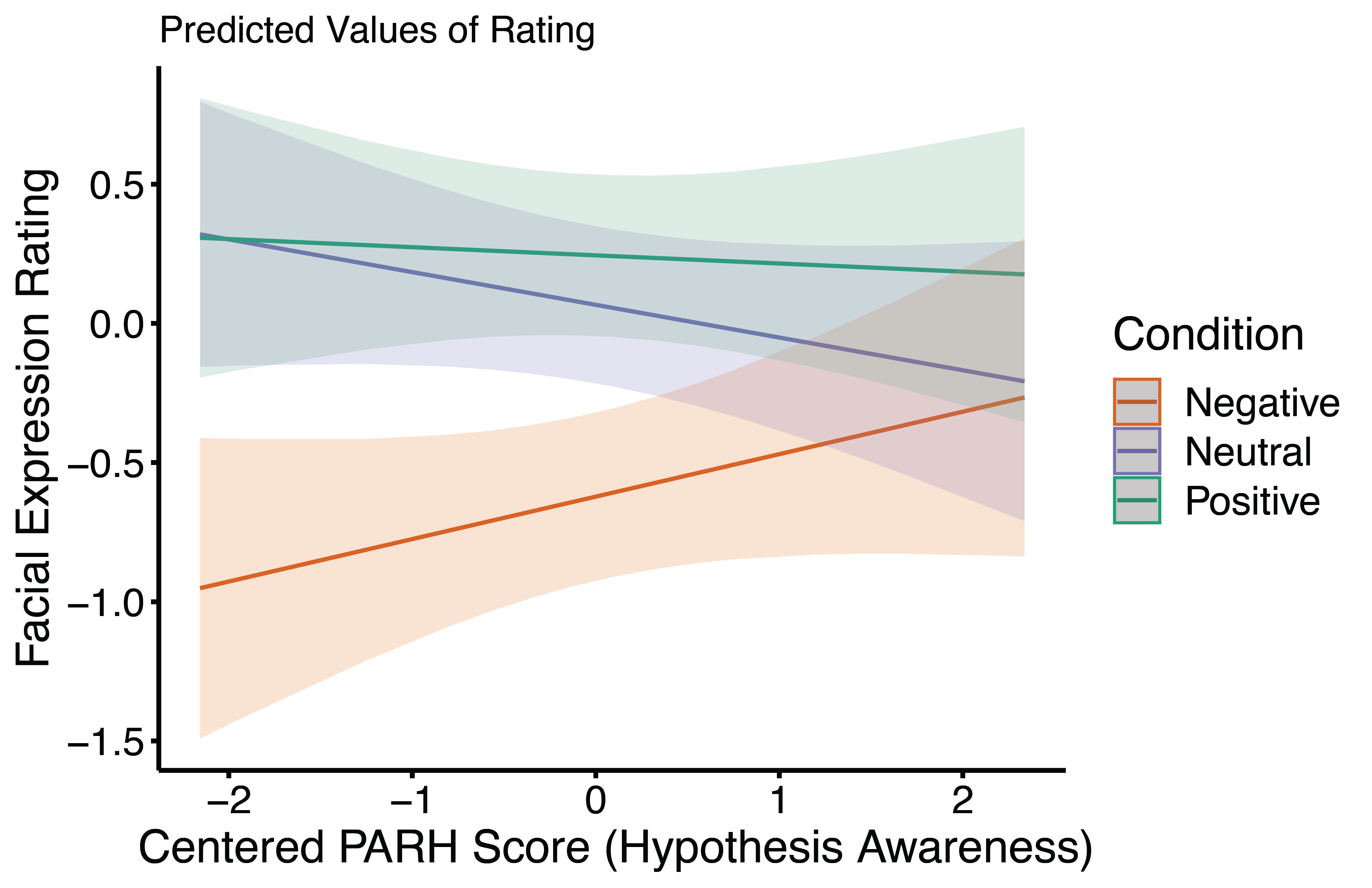

Supplement: nsaf027_Supp [file nsaf027_supp.zip › scan-24-286-File010.png]
